# Inferring past demography and genetic adaptation in Spain using the GCAT cohort

**DOI:** 10.1038/s41598-025-98272-w

**Published:** 2025-04-24

**Authors:** Jorge Garcia-Calleja, Simone A. Biagini, Rafael de Cid, Francesc Calafell, Elena Bosch

**Affiliations:** 1 Institute of Evolutionary Biology (UPF-CSIC), Department of Medicine and Life Sciences, Universitat Pompeu Fabra, 08003 Barcelona, Spain; 2 Department of Archaeology and Museology, Masaryk University, Brno, Czech Republic; 3 Center of Molecular Medicine, Central European Institute of Technology, Masaryk University, Brno, Czech Republic; 4 Genomes for Life-GCAT lab, CORE Program, Germans Trias i Pujol Research Institute (IGTP), 08916 Badalona, Spain; 5 Grup de REcerca en Impacte de les Malalties Cròniques i les seves Trajectòries (GRIMTra), Germans Trias I Pujol Research Institute (IGTP), 08916 Badalona, Spain

**Keywords:** Post-admixture selection, Spanish population, Selection scan, Positive selection, Human adaptation, Demography, Population genetics, Evolutionary biology

## Abstract

Located in the southwestern corner of Europe, the Iberian Peninsula is separated from the rest of the continent by the Pyrenees Mountains and from Africa by the Strait of Gibraltar. This geographical position may have conditioned distinct selective pressures compared to the rest of Europe and influenced differential patterns of gene flow. In this work, we analyse 704 whole-genome sequences from the GCAT reference panel to quantify gene flow into Spain from various historical sources and identify the top signatures of positive (adaptive) selection. While we found no clear evidence of a 16th-century admixture event putatively related to the French diaspora during the Wars of Religion, we detected signals of North African admixture matching the Muslim period and the subsequent Christian Reconquista. Notably, besides finding that well-known candidate genes previously described in Eurasians also seem to be adaptive in Spain, we discovered novel top candidates for positive selection putatively associated with immunity and diet (*UBL7*, *SMYD1*, *VAC14* and *FDFT1*). Finally, local ancestry deviation analysis revealed that the MHCIII genomic region underwent post-admixture selection following the post-Neolithic admixture with Steppe ancestry.

## Introduction

During the Out-Of-Africa migration^1^, a small portion of modern humans dispersed from their native habitat in Africa and started to occupy a diverse range of new environments^2^. This major migratory event, along with subsequent local expansions around the world, explains the overall reduced genetic diversity of non-African compared to African populations. Nevertheless, the diversity of ecosystems colonized by modern humans also drove phenotypic and genetic variation across populations, providing a suitable model for studying local adaptation^3–8^. In recent years, numerous statistical tests have been developed to identify regions of the genome that have experienced positive (adaptive) selection^9–15^. Relying on the theoretical framework provided by population genetics^16,17^, these genome-wide selection scans can be used to pinpoint patterns of genetic variation that deviate from neutral expectations while being compatible with selection at different timescales^15^. Analysis of genomic footprints of selection across human populations in various biomes and under diverse selective pressures has shed light on the origin and genetic basis of adaptive traits, such as lactase persistence^18^, light skin pigmentation in response to lower UV radiation^19^, and high-altitude adaptation^20^, among others. Additionally, geographically restricted adaptive responses to local pathogens and other environmental stresses may explain population differences in some immune-related phenotypes and other disease-related traits such as drug response, hypertension, obesity, and diabetes, among many others^8,21^.

The Iberian Peninsula, located in the southwestern corner of Europe, is bordered by the Mediterranean Sea and the Atlantic Ocean and lies just ~ 13 km away from the North African coast at the narrowest point of the Strait of Gibraltar. Reconstructing the past demography and genetic structure of the Spanish population within the Iberian Peninsula has been challenging. However, recent advances in population genetics, combined with the availability of modern sequencing data^22–25^ as well as ancient DNA datasets^26,27^, have significantly enhanced our understanding of its population dynamics and genetic diversity over time. Like other European regions, the Iberian Peninsula has experienced genetic influence from various human populations arriving from the Levant and the Caucasus^28^. However, it differs in key ways from other parts of Europe. During the glacial period, the peninsula served as a refuge for western hunter-gatherers (WHG), who later also contributed to the genetic diversity of other hunter-gatherer populations across Europe. WHG ancestry is also traceable in Iberian Neolithic farmers, pointing to admixture events between Anatolian farmers and local hunter-gatherers^27^. Proximity to Africa has also contributed to genetic differences between the Iberian Peninsula and the rest of continental Europe^29^. Previous analyses of ancient DNA data have shown that Iberian individuals with high North African ancestry date back as early as 4000 years ago (I4246 from Camino de las Yeseras)^26^. However, these early North African contributions had a limited impact on the overall Iberian genetic pool. In contrast, the ancient DNA record^26^ shows that during the Muslim rule of the Iberian Peninsula (eighth-fifteenth centuries CE), the proportion of North African ancestry in Iberian individuals was higher than it is today^30^. Currently, the varying levels of Northern African admixture are probably the main factor explaining the west-to-east genetic differentiation observed within the Iberian Peninsula^31^. Notably, the availability of a new French dataset^32^ will allow us to explore the genetic impact of an important, yet lesser-known migration: the French diaspora triggered by the Religion Wars in the late sixteenth century^33^, which should be particularly notable in Eastern Iberia (Aragon, Catalonia, Valencia)^33–36^, where it amounted to up to one quarter of the local population^36^. Finally, the geographical structure of genetic diversity in Spain has implications for traits of clinical relevance^37^.

Thanks to its inclusion in the 1000 Genomes dataset^38,39^, the Spanish population has been extensively analysed through genome-wide scans of positive selection. However, no Spanish-specific signal has been reported to date. Here, to explore signatures of recent positive selection in the Spanish population, we take advantage of an exceptional dataset comprising 785 whole genomes from residents of Catalonia, sequenced at 30X coverage as part of the GCAT|Genomes for Life cohort^40–42^. Given Catalonia’s recent history of significant migratory inflows, particularly from other parts of Spain, notably Andalusia, we hypothesize that the GCAT dataset could serve as an appropriate proxy for the broader Spanish population. This approach differs from recent work focused on the microgeographical structure of autochthonous samples from the Catalan Pyrenees^43^. Moreover, the large sample size and high sequencing coverage of the analyzed dataset should provide enhanced statistical power to detect positive selection, including Spanish-specific signals of recent selective sweeps^15^. In this context, we first investigated the genetic structure and potential influence of gene flow from various external population sources, both ancient and present-day. We then applied the SDS (singleton density score)^15^ and XP-EHH (cross-population extended haplotype homozygosity)^10^ methods to detect signals of adaptive selection over a broad timeframe (up to 30,000 years ago). This approach allowed us to identify selection signals for novel as well as known candidate genes, which could be due to either our higher statistical power or gene specificity to southwestern Europe. A Catalan version of the article can be found at: 10.5281/zenodo.15261991.

## Results

### Genetic structure and demography

**Fig. 1:**
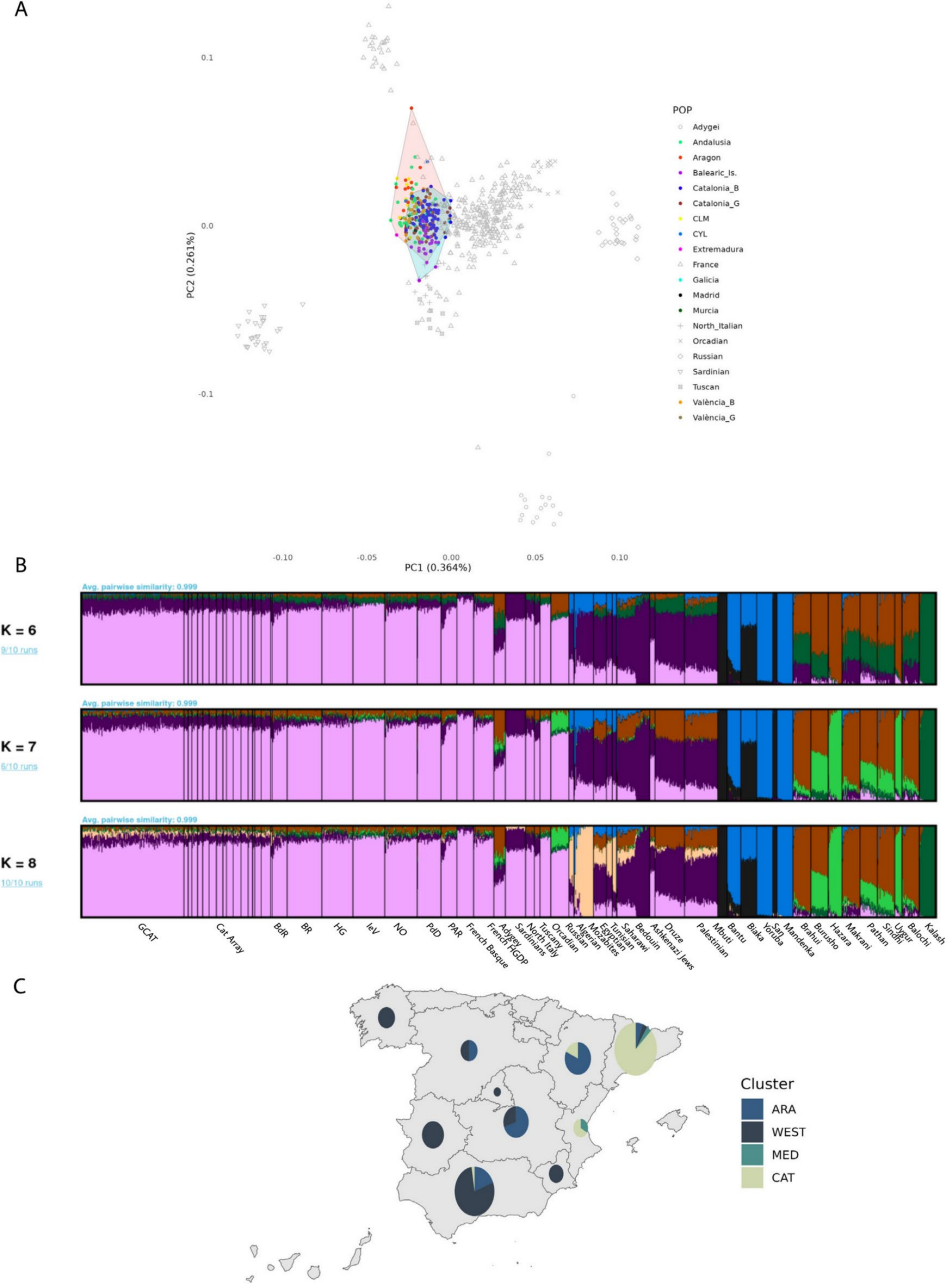
Genetic structure of the GCAT dataset. (**A**) Principal component analysis (PCA) performed with 141,849 SNPs and 141 GCAT samples from individuals whose four grandparents were all born in the same autonomous community within Spain. Each geometric point represents an individual from a particular geographical region. Reference populations were compiled from various sources, covering Catalonia, the Balearic Islands, and the Valencia Community in Spain^44^, France^32^, and the Human Genome Diversity Panel^92^; for additional population details, see Supplementary Table S1. The red polygon encloses all samples from the GCAT dataset, whereas Spanish samples from Biagini et al.^44^ lie within the blue polygon. Abbreviations: CLM, Castilla La Mancha; CYL, Castilla y León. (**B**) Admixture analysis of the GCAT dataset at the lowest cross-validation errors (K = 7, followed by K = 8 and K = 6) using reference populations from Catalonia, the Balearic Islands, and the Valencia Community in Spain^44^, France^32^, North Africa^93^ and the Human Genome Diversity Panel (HGDP)^92^; for additional population details, see Supplementary Table S1. (**C**) Map showing the fraction of GCAT samples clustered into haplotype-based groups defined with fineSTRUCTURE, when silencing external populations. Pie charts are centered to the autonomous communities where these samples belong. Abbreviations: CAT: Catalonia. ARA: Aragon. MED: Mediterranean. WEST: West-Central Spain.

When we explored the GCAT dataset (n = 704; see details in materials and methods) in the context of the five major geographical regions of the 1000 Genomes Project (1000 GP) (i.e., AFR, AMR, EUR, SAS, and EAS) using principal component analysis (PCA), all GCAT samples clustered within the EUR group (Supplementary Fig. S1). When the PCA was restricted to the 1000GP European populations (i.e., IBS, TSI, CEU, GBR, and FIN), GCAT individuals clustered closely with the Iberians (IBS) and Tuscans (TSI) (Supplementary Fig. S2). In a broader Mediterranean context, and restricting the GCAT subjects to those with grandparents originating from the same Spanish region (n = 141), they clearly overlapped with several other European populations, including Catalan, French, and other Europeans (Fig. 1A; Supplementary Table S1). Focusing more specifically on samples from Spain and France, these GCAT individuals clustered with Catalonian, Balearic and Valencian samples^44^ (Supplementary Fig. S3). Incorporating ancient data from the three major Mesolithic and Neolithic components that have shaped present-day European populations—western hunter-gatherers (WHG), early European farmers (EEF), and early Steppe nomads (ENS)^45^—showed that the affinity of GCAT individuals to EEF is closer compared to other European populations such as Orcadian, French, or Italian, although not as close as Sardinians (Supplementary Fig. S4). ADMIXTURE analysis with contemporary neighbouring populations confirmed a major European ancestry component within the GCAT dataset, accompanied by two minor ancestry components present mostly in Middle Eastern and North African populations at K = 7 (lowest cross-validation error followed by K = 8 and K = 6) (Fig. 1b, Supplementary Fig. S5). At K = 8, a further component, mostly characterizing Mozabites but also present in other North African populations and in Palestinians, emerged consistently in all the Spanish samples as well as in those from Provence (Bouches-du-Rhône, BdR) and Sardinia. Thus, while the residents of Catalonia sampled by GCAT mostly show a typical continental European genetic profile, they also contain a small proportion of North African (mean: 0.0442, SD: 0.0023) and Middle Eastern ancestries (mean: 0.1010, SD: 0.0019).

We next explored for haplotype-based clustering of individuals within the same regional GCAT dataset (n = 141) and neighbouring Mediterranean populations using fineSTRUCTURE (Supplementary Fig. S6A). The Spanish population splits in two main branches: East and West (Supplementary Fig. S6b). The East branch is further divided into two clusters, one with samples from Catalonia and Valencia (CAT, as shown in Fig. 1C) and the other with samples from Aragon (ARA). The West branch is subdivided into Eivissa (Ibiza, not shown in Fig. 1C), a Central West cluster comprising samples from Andalusia, Castile, and Extremadura (WEST), and a third cluster with some samples from Valencia (MED). The geographical distribution of these haplotype-based clusters shows an east-to-west gradient of genetic differentiation, consistent with previous analyses and the historical process of the Spanish Reconquista^30,31^. Additionally, four main branches were obtained for France (Supplementary Fig. S6B): French Basques, northern samples from Paris and Alsace, southern samples from Provence, Dordogne and the Pyrenees, and Breton samples from Rennes, the latter having been previously shown to be genetically distinct from other French populations^32,46^. The North African samples were separated into a West and an East group (Supplementary Fig. S6A), the latter having a higher Middle Eastern component, as expected^29^. Subsequently, admixture events for the East and West genetic clusters of the Spanish population were formally inferred using fastGLOBETROTTER^47^. Both clusters revealed a single admixture event between a southwestern European source (source 1, primarily consisting of samples from Provence, Dordogne, the Pyrenees, and Brittany) and a minor African-like source (source 2). The proportions were 98% and 2% for the West Spain cluster and 96% and 4% for the East Spain cluster, respectively (Supplementary Fig. S7A). Although fastGLOBETROTTER initially suggested two admixture events in the West Spain cluster, this pattern proved non-robust after bootstrapping. Interestingly, for the West Spain cluster, the West North African cluster was inferred as a surrogate population in both sources 1 and 2, possibly reflecting ancient gene flow between the Iberian Peninsula and the Maghreb. Additionally, French Basques were identified as a surrogate population in sources 1 and 2 for both clusters, pointing to a Basque admixture event, consistent with previous reports^30^. The inferred dates for these admixture events differ slightly between clusters, being ~ 1153 AD (CI 95%: 1083–1242 AD) for East Spain, and ~ 1211 AD (CI 95%: 1119–1287 AD) for West Spain, assuming 29 years per generation (Supplementary Fig. S7B).

### Finding signatures of selection

**Fig. 2:**
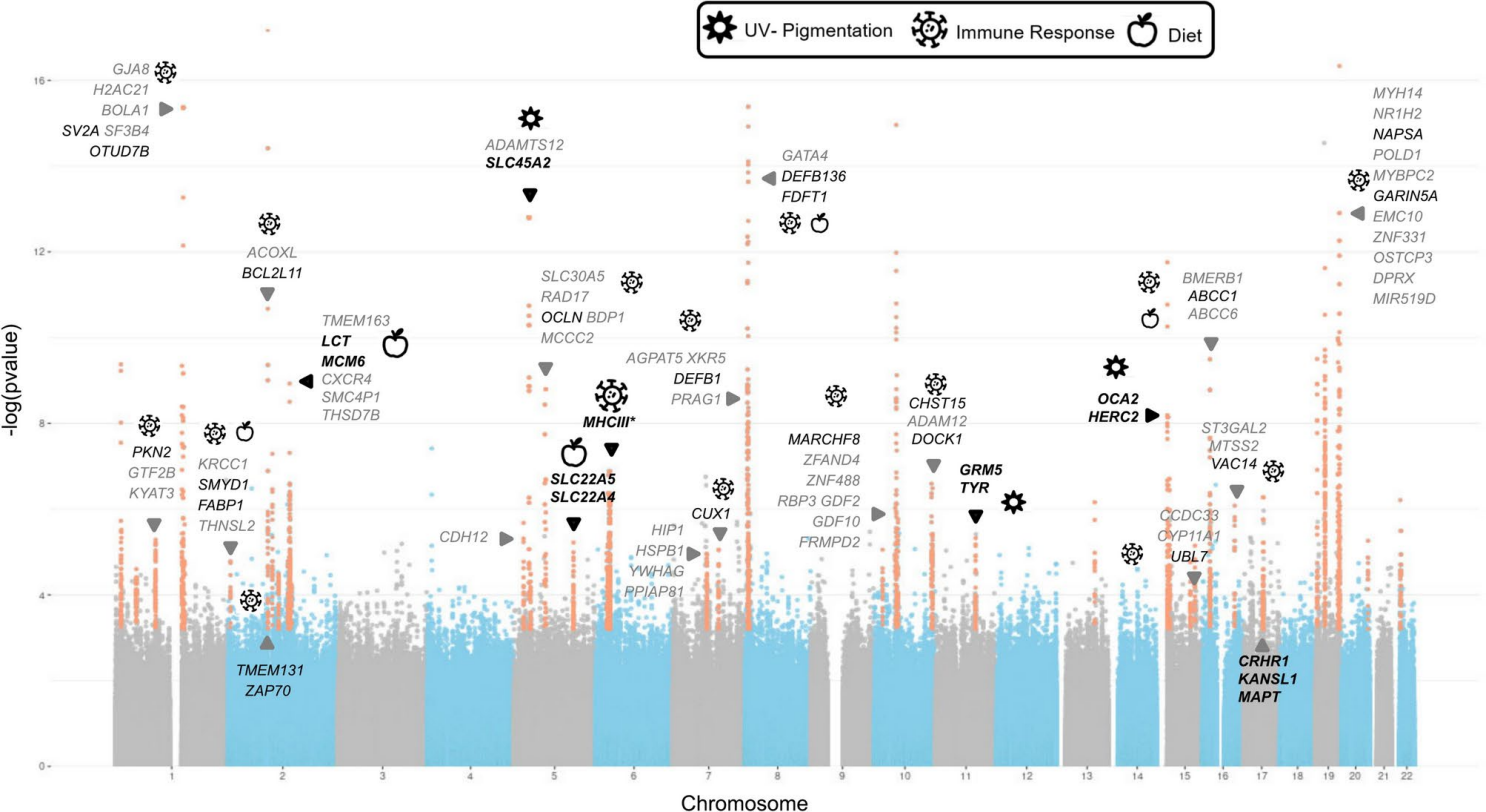
Manhattan plot of signatures of recent positive selection in the GCAT dataset. The y-axis indicates the –log10 (*p*-value) of the singleton density score (SDS) statistic calculated from the WGS data of 704 Spanish individuals in the GCAT dataset. The 40 selection peaks highlighted in orange represent SNPs above the top 99.99% SDS values accompanied by at least 10 SNPs above the 99.995% SDS values within a 1 Mb region (for details on SDS values, genes, and SNP annotations, see Supplementary Tables S2-S5). Genes associated with plausible adaptive biological functions are shown in black. Well-known candidate genes previously associated with adaptive evolutionary traits are in bold.

Signals of selection were initially explored using SDS^15^ in the entire GCAT dataset (n = 704) to investigate very recent positive selection events. Additionally, we employed XP-EHH^10^ to detect high-frequency haplotypes that have been recently swept to relatively high frequencies in the GCAT dataset, using the YRI population as a reference (Figs. 2 and S8). After annotating the corresponding genes and SNP variants for each selection peak (for details, see Materials and Methods), we further investigated putative candidate variants for selection using iSAFE^12^ and CLUES^14^ (Supplementary Tables S2–S9). As expected, most of the candidate variants confirmed by CLUES and initially identified through SDS exhibit lower allele frequencies and correspond to more recent selective sweeps compared to those identified by XP-EHH (Supplementary Figs. S9-S25). Similarly, whereas 12 out of the 40 SDS selection peaks described in the GCAT dataset comprise well-known candidate regions for positive selection previously identified in Europeans (Supplementary Table S3), the majority of signals detected with XP-EHH in GCAT correspond to selective sweeps already described in both Europeans and Asians when compared with the YRI population (Supplementary Table S7). As discussed below, a significant number of candidate regions for positive selection identified in the GCAT dataset are related to lighter skin pigmentation, diet, and immune response (Table 1).

**Table 1:** Top candidate genes for positive selection in the GCAT dataset.

Function	Genes	SNP	Type	Method	LogLR	s
Pigmentation	*SLC45A2*	rs183671	Intronic	SDS	8.06	0.00427
	*OCA2-HERC2*	rs7183877	Intronic	SDS	8.75	0.00104
	*KITLG*	rs556861	Intronic, non coding transcript	XP-EHH	6.53	0.00342
	*GRM5-TYR*	rs7119749	Intronic	SDS	9.81	0.00371
	*SLC12A1-DUT*	rs9920281	Intronic	XP-EHH	5.39	0.01865
	*MLPH-RAB17*	rs10176842	Intronic	XP-EHH	5.02	0.00255
Diet	*MCM6-LCT*	rs4988235	Intronic	SDS	22.52	0.00862
	*SLC22A4*	rs1050152	Missense	SDS	11.68	0.00503
	*ABCC1*	rs212086	Intronic	SDS	3.71	0.00430
	FDFT1*	rs1296025	Intronic	SDS	4.72	0.00576
	*CYP3A4*	rs2404955	Downstream gene variant	XP-EHH	7.90	0.00383
Immune response	UBL7*	rs750607	Downstream gene variant	SDS	9.46	0.00361
	SMYD1*	rs35662596	Intronic	SDS	6.12	0.01294
	PRAG1*	rs55852693	Downstream gene variant	SDS	5.25	0.00413
	*PBX2*	rs204991	Upstream gene variant	SDS	6.68	0.00774
	*TMEM232*	rs10038763	Intronic, non coding transcript	XP-EHH	9.24	0.00374
	MTSS2-VAC14*	rs11075777	Intronic	SDS	3.58	0.00222

CLUES estimates of the selection coefficient (s) and the likelihood of the selection inference (LogRT) in the GCAT dataset are indicated in the two last columns. * Newly identified candidates in this study. For additional details, see Supplementary Tables S5 and S9.

#### Selection in pigmentation genes

Three of the top SDS peaks identified in the GCAT overlap with well-known candidate genes for selection related to lighter skin pigmentation in Europeans: *SLC45A2*^48^, *OCA2*-*HERC2*^11^, and the *GRM5-TYR*^11^ region (Table 1, Fig. 2). In *SLC45A2* we found strong evidence for selection acting on variant rs183671, which is associated with several skin/hair/eye pigmentation traits according to the NHGRI-EBI Catalogue of GWAS (https://www.ebi.ac.uk/gwas/; Supplementary Table S2). Using CLUES to estimate the past frequency trajectory of the selected derived allele, we detected a selective sweep within the last 500 generations (Supplementary Fig. S9). In the *OCA2* genomic region, we estimated strong evidence for selection at the *HERC2* intronic variant rs7183877, also associated with skin, hair, and eye pigmentation (Supplementary Table S2, Supplementary Fig. S10). Another SDS peak potentially related to lighter skin pigmentation comprised a putatively selected intronic variant in the *GRM5* gene (rs7119749, Supplementary Fig. S11). This variant is located upstream of the *TYR* gene, which encodes the enzyme involved in the first step of melanin synthesis. In the XPEHH analysis comparing YRI and GCAT, we detected up to three additional candidate regions that may be related to lighter skin pigmentation and adaptation to lower UV radiation, as described in previous studies^49^: *SLC12A1*-*DUT* (located near the *SLC24A5* gene), *MLPH*-*RAB17*, and the *KITLG* region (Table 1, Supplementary Figs. S12-S14). The *SLC24A5* gene encodes a sodium-calcium exchanger associated with pigmentation in zebrafish and humans, possibly by facilitating ion transport in melanosomes^50,51^, whereas the melanophilin gene (*MLPH*) plays a key role in melanosome transport and prostate cancer susceptibility^52,53^. *KITLG* encodes the ligand of the tyrosine-kinase receptor, which has been shown to influence pigmentation by regulating melanocyte proliferation and melanin distribution^54^.

#### Selection in genes related to diet

Two selection peaks obtained with SDS contain well-known candidate regions for dietary adaptation: *LCT-MCM6* and the region containing the *SLC22A4* and *SLC22A5* genes (Fig. 2, Table 1). As expected for a European population, we found robust evidence supporting positive selection for the derived allele of the intronic rs4988235 *MCM6* variant associated with lactase persistence in Europeans. Using CLUES in the genome-wide genealogies inferred from the GCAT dataset, we estimate this selection event to have started within the last 200 generations (Supplementary Fig. S15). In the ergothioneine transporter gene *SLC22A4*, we find strong evidence for positive selection acting on the missense variant rs1050152 (encoding the L503F substitution) within the last 300 generations (Supplementary Fig. S16). This gene is thought to exhibit a selection signal due to adaptation to the low dietary levels of ergothioneine among early Neolithic farmers in the Fertile Crescent^55,56^. Interestingly, the selected variant is associated with reduced expression of the *SLC22A5* gene according to GTEX (Supplementary Table S2), resulting in lower levels of the *OCTN2* carnitine transporter, which is important for transportation and oxidation of fatty acids in mitochondria^57^. Another selection signal identified with SDS possibly related to adaptive detoxification was observed in the *ABCC1* gene in chromosome 16, where we detected positive selection acting on the intronic variant rs212086, although with lower confidence (Table 1, Supplementary Fig. S17). Notably, *ABCC1* encodes a well-known multidrug resistance protein (MRP1), which plays a role in the biliary detoxification of various anti-cancer drugs^58^ and has previously been detected under positive selection in the CEU population^59^. Additionally, we found moderate evidence for positive selection acting on the downstream gene variant rs2404955 in the *CYP3A4* gene (Supplementary Fig. S18), which is similarly involved in detoxification, as well as in the metabolization of numerous therapeutical drugs^60^*.*

#### Selection in immune response genes

A high proportion of the candidate regions for positive selection identified in the GCAT dataset contain genes putatively related to the immune response (Fig. 2, Table 1). For example, a large candidate region detected with SDS, spanning over ~ 1.474 Mb on chromosome 6, comprises the MHC III region, which includes multiple immune-related genes such as *HCG20*, *HCG21*, *AIF1*, *GPANK1*, *ABHD16A*, *LY6G6F*, *C2*, and *PBX2*. Using CLUES to analyse several putative functional variants in this region, we detected moderate evidence of selection acting within the last 150 generations on rs204991, a SNP upstream of *PBX2* (Supplementary Fig. S19). According to GTEX, this SNP strongly influences the expression of complement components 4A and 4B in various tissues. Additionally, using the XP-EHH statistic, we replicated a previously known selection signal at the *TMEM232* gene in Eurasians^61^, which has been shown to promote inflammatory response in atopic dermatitis^62^. Among several candidate variants in the *TMEM232* region, the strongest evidence for selection was found for the non-coding transcript exon SNP rs10038763 (Supplementary Fig. S20).

#### New candidates for adaptation

Our analysis of the top SDS signals in the GCAT (Fig. 2) also revealed several previously undocumented candidate regions and new putatively selected variants (Table 1). For instance, we found moderate evidence of selection within the last 6000 years for the *SMYD1* intronic SNP rs35662596 (Supplementary Fig. S21), which reaches its highest global frequency in the IBS population (14%). Notably, mutations in *SMYD1* can lead to the absence of the AnWj antigen on the surface of red blood cells^63^, a receptor for *Haemophilus influenzae*^64^. The amount of AnWj correlates with the ability of *H. influenzae* to adhere to epithelial cells^65^. We also identified moderate evidence of selection acting on the *FDFT1* intronic SNP rs1296025 (Supplementary Fig. S22), whose highest allele frequencies in the 1000 GP are found in CEU (17%), GBR (17%) and IBS (21%) populations. Notably, *FDFT1* encodes the first specific enzyme in the cholesterol biosynthesis pathway^66^ and is a downstream target of the fasting response^67^, whereas rs1296025 has been associated with non-HDL cholesterol levels^68^. Another novel candidate for positive selection lies within the *UBL7* region, harbouring the regulatory variant rs750607 (Supplementary Fig. S23). According to ensembl^69^, this SNP is associated with differential expression of several genes across the region, including Semaphorin 7A (*SEMA7A)* in neutrophils, which is involved in immune response, inflammation^70^, and the regulation of NK cells^71^, T cells^72^, and mastocites^73^. Within the *PRAG1* region, weaker evidence for selection was detected for the downstream gene variant rs55852693, which is predicted to disrupt a transcription factor binding site (Supplementary Fig. S24) and has been associated with enjoyment of spicy food^74^. Although allele frequencies for rs55852693 are similar across all European populations within the 1000GP, which suggests it may not correspond to a Spanish-specific adaptation, *PRAG1* has also been identified by a selection scan of an admixed Brazilian population, in a genomic region enriched for the Native American ancestry^75,76^. Finally, within the *MTSS2-VAC14* SDS peak, we found weak evidence of positive selection acting on the *MTSS2* intronic rs11075777 SNP (Supplementary Fig. S25). The selected allele is an eQTL for the *VAC14* gene, which plays a role in susceptibility to bacteremia^77,78^. However, given that its frequency is approximately 50% across all European populations in the 1000 GP, it might also represent a broader European signature of selection.

### Local ancestry deviations

**Fig. 3:**
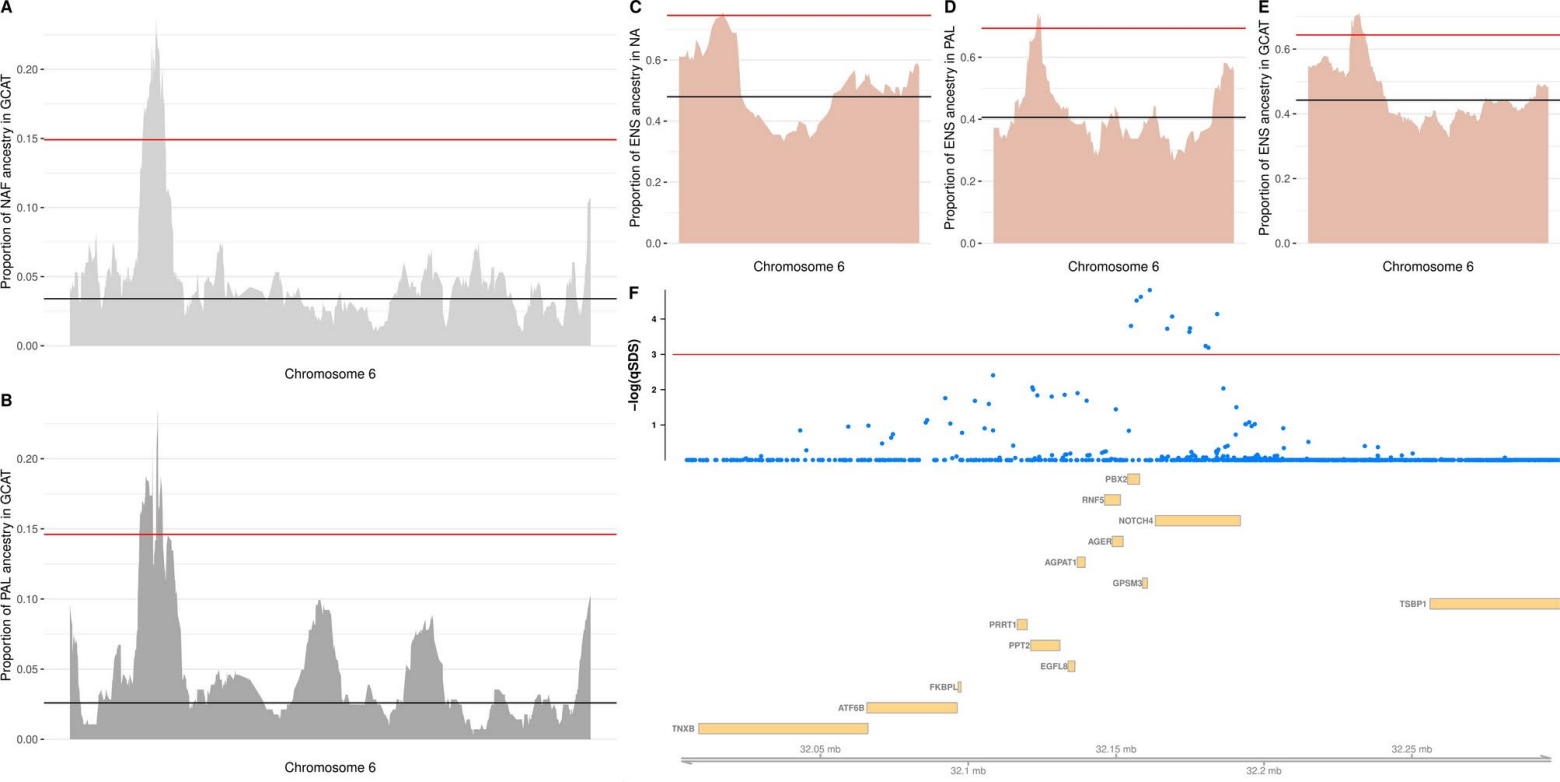
Post-admixture selection in the MHC III region. (**A**) Proportion of North African (NA) ancestry across chromosome 6 in the GCAT dataset. (**B**) Proportion of Palestinian (PAL) ancestry across chromosome 6 in the GCAT dataset. Black line, mean genomic LAD; red line, significant LAD (4.42 SD). (**C**) Proportion of early Steppe nomad (ENS) ancestry across chromosome 6 in North Africa. (**D**) Proportion of ENS ancestry in Palestinians. (**E**) Proportion of ENS ancestry in the GCAT dataset. Black line, mean genomic LAD; red line, significant LAD (3 SD). (**F**) Transformed SDS scores after FDR correction, and putative candidate genes overlapping the LAD region detected in chromosome 6. For details and genomic positions, see Supplementary Table S10. Red line indicates a statistical significance cut off of 0.05.

Next, we explored the genomic regions presenting local ancestry deviations (LAD) associated with specific ancient or modern ancestries that overlap with selection peaks identified via SDS or XP-EHH, as these regions could potentially represent cases of post-admixture selection. In the regional GCAT subset (n = 141), modern LADs were assessed using proxy samples from North Africa for North African ancestry, Palestine for Middle Eastern ancestry, and Southern France as the closest European population to represent the autochthonous background of the GCAT (for details, see Materials and Methods). We identified three overlapping LAD regions (SD > 4.42) on chromosome 6, pointing to an excess of North African and Middle Eastern ancestries, overlapping with three SDS peaks of positive selection (Supplementary Table S10). Two of these SDS peaks displayed LAD for both North African and Middle Eastern ancestries and overlapped with several genes in the MHC III region, including the upstream *PBX2* SNP rs204991, which regulates *C4A* and *C4B* expression (Fig. 3).

To investigate LAD regions resulting from extensive admixture events during the Mesolithic and Neolithic periods in Europe, we leveraged the Mesoneo Dataset^45^, using the EEF, WHG, and ENS samples as proxies to estimate the corresponding ancient ancestry proportions of each GCAT individual (n = 704). This analysis revealed an overall pattern similar to that observed in Bronze Age Iberian individuals (Supplementary Fig. S26). Using a strict cut-off of 4.42 SD^79^, no LAD regions were found for these ancient ancestries, but when considering a more lenient cut-off of 3 SD, we detected up to five regions with an excess of WHG ancestry, two with ENS ancestry, and one with EEF ancestry (Supplementary Table S10 and Supplementary Figs. S27-S28). Among these LAD regions, only three overlapped with the top 40 signatures of selection detected with SDS in the GCAT dataset. Notably, these included the *LCT-MCM6* peak (Supplementary Fig. S29) as well as the third SDS selection peak on chromosome 6 comprising the MHC III region (Fig. 3), both displaying ENS ancestry deviation. The third ancient LAD overlapping an SDS peak contains a cluster of zinc finger genes on chromosome 19 and shows an excess of WHG ancestry (Supplementary Fig. S29). Interestingly, although not presenting recent signatures of positive selection in the GCAT dataset, the only LAD associated with EEF ancestry comprised the *FADS2* gene. This gene has been previously identified as a target of strong selection across diverse ancestries, including Eastern and Western hunter-gatherers as well as Anatolian farmer populations^6^.

## Discussion

Our analyses demonstrate that the GCAT cohort not only captures the genetic characteristics of a typical European population but also serves as a robust proxy for the general Spanish population within the Iberian Peninsula. As our samples covered regions across Spain, we were able to detect the well-documented genetic contribution from North Africa, which is differentially structured between eastern and western Iberia^30^. However, we failed to find the contribution to the current Spanish gene pool of the French diaspora linked to the 16th-century Wars of Religion. Given the short genetic distance between NE Iberia and S France (which mirrors the linguistic and surname similarities that facilitated the assimilation of these newcomers) and the smaller sample size of individuals that could be assigned to a particular region given by the birthplace of their four grandparents, our design may have been underpowered to capture this genetic contribution.

Leveraging the enhanced statistical power provided by the large sample size of the GCAT dataset, we were able to identify new candidate genes under positive selection and replicate several well-known cases of adaptive selection related to pigmentation, diet, and the immune response, previously described using different selection methods. As expected, the candidate regions we replicated using the XP-EHH statistic generally correspond to selection signatures shared across various non-African populations, probably reflecting common environmental pressures following the Out-Of-Africa migration. In contrast, the signatures identified with the SDS statistic are predominantly shared with other European populations or, in some cases, are specific to the Spanish population, indicating more recent and localized selective events. Thus, the combined use of SDS and XP-EHH in the GCAT enabled us to uncover a comprehensive set of genomic selection footprints, shaped by the diverse evolutionary pressures experienced by the ancestors of contemporary Spanish populations across different time periods. While many of the identified selection signatures and well-known candidates are shared with other European populations, differences in the timing and selection strength were clearly observed in the GCAT dataset. These differences may stem from varying demographic histories, external influences, and environmental conditions. For instance, the selection coefficient estimated here for rs4988235 at *LCT* (s = 0.00862) is slightly lower than (though consistent with) previous estimates (s = 0.0194, s = [0.01019, 0.01056])^6,80^. Moreover, the lactase persistence allele seems to have emerged earlier in northern Europe than in the Iberian Peninsula^26^ and its frequency is lower in the Spanish population. Conversely, the current frequency of the selected rs1050152 allele at *SLC22A4* seems to be higher in the Spanish population (45%) compared to other European populations in the 1000GP (36–41%). This difference may be attributable to the higher EEF ancestry component inferred in the GCAT dataset. Interestingly, while Irving-Pease et al.^6^ inferred that selection on variant rs1050152 ceased ~ 1500 years ago, our analysis indicates a continuous rise in allele frequency until very recent times, consistent with findings by Mathieson et al.^81^. Similar high-resolution studies of positive selection in Italy have revealed differential adaptive signatures between northern and southern Italian populations, although the most plausible mechanism involved is probably varying levels of genetic drift^82^. Unfortunately, the GCAT cohort does not include enough individuals with all four grandparents sharing the same birthplace to perform a robust latitudinal and environmental exploration of positive selection in Spain.

Like other continental European regions, the Iberian Peninsula has been influenced by the cultural transitions and demographic changes of the Holocene. The shift from hunter-gatherer societies to Neolithic agricultural systems, followed by the arrival of pastoralist-nomadic groups, not only transformed the genomic landscape of the Iberian population but also probably introduced new selective pressures, leaving distinct genomic imprints. Moreover, as these incoming populations were likely well-adapted to their respective lifestyles and cultural practices, they may have also contributed adaptive variants to the Spanish gene pool through admixture. Our analysis of the GCAT dataset shows three regions with significant LAD towards the WHG and ENS components, overlapping with recent signals of selection. Interestingly, the LAD region on chromosome 19 (Supplementary Fig. S29), enriched for the WHG ancestry component, harbours a cluster of zinc finger genes. However, the exact function and possible adaptive phenotypes associated with these zinc finger genes remain unknown and require further research to enhance our understanding of pre-Neolithic genetic adaptations in WHG populations. Additionally, LAD analysis showed that the SDS selection peak at the MHC III region was significantly enriched for ENS ancestry. When only contemporary populations were considered, this region displayed parallel enrichment for North African and Middle East ancestry components. Notably, the two populations used as proxies for such external components also displayed significant LAD for the ENS ancestry component, suggesting a shared selective pressure potentially related to domestication and zoonotic transmissions^83–85^. It should be noted that the high genetic diversity of the HLA region can potentially confound local ancestry inference methods, as observed in research on post-admixture selection in Latin American populations, which showed an excess of African ancestry at the HLA signals^86^. Nevertheless, this bias is not expected to affect the comparison with ENS in our study. Finally, the *LCT-MCM6* region also displayed significant deviation for ENS ancestry in the GCAT dataset. This pattern agrees with previous reports that the signatures of positive selection on *LCT* can be traced to Steppe ancestry^6^.

While our study provides valuable insights, it is important to acknowledge some inherent limitations and potential biases. First, the GCAT cohort exhibits a geographic imbalance, with an overrepresentation of individuals from southern and eastern Spain compared to northern and western Spain. This asymmetry limits our ability to thoroughly analyse the different periods of North African admixture across the Iberian Peninsula, as the timing of the transition from Muslim to Christian rule varied significantly between regions. As a result, regional differences in admixture patterns arising from distinct historical interactions with North African populations may not have been fully captured. Second, the characterization of top candidate regions for selection (or selection peaks) relies on two user-defined parameters, which could introduce biases: namely, the length of the window used to detect high-scoring alleles and the number of alleles with genome-wide significant statistical values required to classify a genomic region as part of a peak. These arbitrary thresholds may influence both the identification and interpretation of selection signals detected in genome-wide scans performed using SDS and XP-EHH. To address this, we aimed to validate all candidate regions for selection using iSAFE, enabling the identification of favoured alleles^12^. Additionally, we applied CLUES to visualize past allele frequency trajectories of each putative selected allele and to estimate the corresponding selection coefficient and likelihood of positive selection^15^. Finally, the low sample size of populations used as North African proxies may have limited the power of our LAD analyses and admixture time estimations. A more comprehensive understanding of the North African admixture process could be achieved by incorporating high-coverage whole genome data from Northern African populations, particularly Amazigh groups, who played a major role in the Islamic Conquest of Iberia, as documented in historical records such as the Muqaddimah by Ibn Khaldūn^87^.

In conclusion, our findings demonstrate that several candidate genes previously identified as adaptive in other parts of Europe were subject to positive selection in the ancestral populations of present-day Spaniards. Additionally, we identified novel candidate genes for positive selection, which may be due to the larger sample size used in our study or their specificity to southwestern Europe. However, these genes should be considered as provisional candidates until the functionality of their genetic variation and evolutionary relevance are thoroughly characterized and understood.

## Materials and methods

### GCAT cohort and genome data processing

VCF files for Illumina 30X Whole Genome Sequences (WGS) of 785 present-day individuals from the GCAT cohort^42^ were obtained from the European Genome-phenome Archive (EGA) under accession number EGAD00001007774. The GCAT cohort was recruited (2014–2018) from residents in Catalonia aged 40–65 with access to the national public healthcare system. It consists of 19,140 registrants. The characteristics of the cohort^40,41,88^ and sequenced dataset are described elsewhere^42^. Complete genome sequences were available for 785 volunteers; of those, registered metadata indicated that 141 had all four grandparents born in the same Spanish region, be it Catalonia or any other one (see details in Supplementary Tables S1 and S11). Quality control and filtering of admixed individuals has been performed previously and are described elsewhere^42^. To focus on biallelic SNPs, BCFtools was used to exclude structural variants and indels. Additionally, 81 GCAT individuals with self-reported non-Caucasian ethnicity were removed (although the meaning of “Caucasian” in the Spanish context is unclear; Supplementary Table S12). The final sample size was therefore 704 (Dataset A).

The VCF files were then lifted over to hg38 using Picard tools^89^ and merged with the 1000 GP Phase 3^39^ using the isec command in BCFtools^90^ (Dataset B). Subsequently, rare variants were removed, SNPs displaying strong Hardy–Weinberg deviations were filtered out, and we pruned for linkage disequilibrium (LD) using PLINK2^91^ in sliding windows of 200 kb, a step size of 25 SNPs, and a square correlation coefficient threshold (r^2^) of 0.5 (–maf 0.05 –max-maf 0.95 –hwe 1e-50 –indep-pairwise 200 25 0.5). At this point, the dataset consisted of 679,677 variants and 3,208 individuals. Principal components analyses (PCA) were performed using the smartPCA tool from the EIGENSOFT package^92^ and the EIGENSTRAT^93^ correction without outlier removal (Supplementary Figs. S7-S2).

#### ADMIXTURE and fineSTRUCTURE analyses

The GCAT dataset was filtered to retain individuals whose four grandparents were all born in the same autonomous community (first-level administrative division in Spain) and merged with available datasets containing suitable proxies for detecting external contributions to the Iberian Peninsula. Accordingly, the 141 samples with all four grandparents from the same autonomous community were initially merged with the HGDP panel^94^ using the same procedure as for Dataset B. Additional SNP array genotyping data from France^32^, Catalonia^44^, and North Africa^95^, were also included. Related samples up to the third degree were not found.

Rare variants were removed, SNPs with strong Hardy–Weinberg deviation were filtered, and LD pruning was performed using PLINK2 in sliding windows of 200 kb, a step size of 25 SNPs, and an r^2^ threshold of 0.5 (–maf 0.05 –max-maf 0.95 –hwe 1e-50 –indep-pairwise 200 25 0.5), retaining 215,178 variants. PCA was conducted using the smartPCA tool from EIGENSOFT package with the EIGENSTRAT correction but without outlier removal. Samples from South America, Oceania, and East Asia were excluded due to their lack of relevance to the ancestry components in GCAT, as revealed in previous studies^24,30^. Thus, the final dataset (Dataset C) consisted of 1,181 individuals (see Supplementary Table S1 for details). Individual ancestries in this pruned dataset were explored with ADMIXTURE 1.3^22^ in 10 runs using the unsupervised mode and tested from K = 1 to K = 12. We used PONG^96^ to plot the ADMIXTURE results.

The unpruned dataset, containing 426,650 variants across 1,181 individuals, was phased using SHAPEIT 4.1.3^97^ and the 1000 GP haplotype reference panel^39^. Subsequently, CHROMOPAINTER^98^ was run in all-versus-all mode for chromosomes 1, 4, 17, and 20 to estimate the switch rate parameter (N_e_) and global mutation rate (M) using 10 iterations of CHROMOPAINTER’s expectation–maximization algorithm (EM). Using these values, we reran CHROMOPAINTER in all-versus-all mode, specifying that all individuals should copy from any other individual for all chromosomes. The fineSTRUCTURE^98^ Markov Chain Monte Carlo (MCMC) method was then applied to assign each individual to a genetic cluster using 1,000,000 burn-in iterations (parameter -x), and 2,000,000 sample iterations (parameter -y) from which we only retained every 10,000th iteration (parameter -z). Additionally, fineSTRUCTURE was rerun using the force file (-F) to fix clusters outside Spain, France, and Italy as continental groups.

The two major inferred Iberian genetic clusters (West and East) were analysed further using fastGLOBETROTTER^47^ in donor-vs-recipient mode, excluding the Spanish genetic clusters (West, East and Ibiza) as donors and considering all the clusters as recipients (except the non-target Iberian clusters). Subsequently, CHROMOPAINTER was run in donor-vs-recipient mode, using only the target Spanish cluster as the recipient. Next, fastGLOBETROTTER was used with the prop.ind: 1 option to infer and date admixture events. To account for disequilibrium patterns that could confound admixture signals, the null.ind: 1 option was enabled. A second fastGLOBETROTTER run was performed to conduct bootstrap analysis and estimated a confidence interval around the inferred admixture date.

#### Ancient ancestry components

To explore the genetic structure of the GCAT dataset in the context of older admixture, we used a publicly available ancient DNA dataset from Allentoft et al.^45^. The genetic clusters inferred by Allentoft et al.^45^ as proxies for the three principal ancient populations that explain the genetic diversity in present-day Europe were used: western hunter-gatherers (WHG), early Steppe nomads (ENS) and early European farmers (EEF). We used a preprocessing approach following recommended guidelines: discarding low coverage individuals, and keeping sites passing 1000G genomic masks, MAF > 0.05 and INFO ≥ 0.8. This filtering strategy resulted in a dataset containing 2,997,159 SNPs. Restricting the analysis to transversion sites only, yielded a dataset with 966,986 SNPs. A PCA was performed using the same procedure and pipeline as for Dataset C. Given the demonstrated accuracy of imputation for ancient dataset relative to modern day sequence data^45^, PCA projection was not applied.

#### Analyses of positive selection

To investigate candidate regions under positive selection in the GCAT dataset, we employed two statistics: (i) the Singleton Density Score (SDS) to identify very recent selection events; and (ii) the Cross Population Extended Haplotype Homozygosity (XP-EHH) to detect selective sweeps where favoured variants have recently reached high frequencies (or fixation) in the GCAT relative to the YRI population.

To compute the SDS, SNPs in Dataset A were polarized based on their ancestral alleles using custom scripts and the Ensembl EPO fasta file (http://May2024.archive.ensembl.org/info/genome/compara/mlss.html?mlss=2006). Singletons were extracted into separate files. Test SNPs were processed by excluding, rare variants (-maf 0.05 -max-maf 0.95) using PLINK, and keeping sites with three genotypes, resulting in a dataset of 5,251,738 sites. Centromeric regions were also withdrawn from the analysis. We treated the observability of each variant as equal. Gamma shapes were inferred using a European population model based on Tennesse et al.^99^ (implemented in the SDS github repository) with a sample size of 1408 chromosomes for the allele frequencies ranging from 0.05 to 0.95 (in 0.01 steps). Raw results were normalized by bins of derived allele frequencies of 0.05–0.95 (0.01 steps) and *p*-values were then computed. SNPs were classified as being in a candidate region for positive selection if they were in the 99.99% quantile of SDS values, accompanied by at least 10 additional variants within the 99.995% quantile in a 1 Mb genomic window.

In the XP-EHH method, we first phased dataset B using SHAPEIT 4.1.3^97^ and the 1000 GP reference panel^39^ and then computed normalized XP-EHH values using selscan v1.3.0^100^. SNPs were considered to be in a candidate region for positive selection if they fell within the 99.99% quantile of XP-EHH values and were accompanied by at least 10 additional variants in the 99.995% quantile within a 1 Mb genomic window.

SNPs in candidate regions for positive selection were functionally annotated using the Ensembl Variant Effect Predictor (VEP^101^) and each region was then manually explored for putative candidate variants (Supplementary Tables S2 and S6). In addition, we ran iSAFE using windows of 400 kb around the SNP with the highest selection signal in each candidate region using the –IgnoreGaps flag and the YRI population from the 1000 GP as the outgroup (Supplementary Tables S4 and S8). Putative candidate variants for selection were further validated using CLUES. For that, we obtained genome-wide genealogies for each site with Relate using previously inferred coalescent times^13^ on a subset of the merged dataset with 1000 GP including all European, Han Chinese, and Yoruban populations. Population size was then estimated using the –threshold 0.5 for the GCAT population to obtain specific coalescences for the GCAT data. Subsequently, we reestimated the genealogy branch lengths using the RelateCoalescentRate (–mode ReEstimateBranchLengths). Ancestral recombination graphs (ARGs) were sampled with the SampleBranchLengths script, assuming generation times of 28 years with 100 samples. Next, we used CLUES^14^ to estimate the selection coefficients (Supplementary Tables S5 and S9) and their corresponding allele frequency trajectories (Supplementary Figs. S9 to S25) using the previously inferred coalescence times and all the European samples from the ancient DNA dataset without excluding non-transversion sites. Selection inference was restricted to the oldest time sampled (i.e. 528 generations in San Teodoro 3 – ST3, from Sicily, Italy^102^).

#### Local ancestry deviations (LAD)

We explored LAD in the GCAT dataset using contemporary external populations and ancient genetic data. To investigate LAD with contemporary external data, we first ran RFmix^103^ on Dataset C using the following flags: -e 5 -n 5 –reanalyze-reference to apply the EM iteration algorithm and correct admixture individuals in the reference populations. As reference samples we used the fineSTRUCTURE inferred genetic clusters of Southern France (SUD, comprising mostly samples from Provence and Dordogne) downsampled to 30 samples, Western North Africa (WNA, comprising Algerian, Tunisian and Moroccan samples from the Lazaridis dataset)^95^, and Eastern North Africa (ENA, comprising Egyptian and Bedouin samples) in one run (Supplementary Fig. S30). To check whether the detected signals could arise from a prior Levantine migration, we repeated the analysis, merging ENA and WNA into a single North African cluster and using the Palestinian (PAL) cluster as a Middle Eastern proxy. The –reanalyze-reference flag was used to account for possible admixture in the reference panel, which is expected in North African populations. To check whether the detected signals arose from a common ancestral source from Neolithic or post-Neolithic times, we repeated the local ancestry inference across the entire GCAT dataset, as well as the North African (ENA and WNA) and PAL clusters, using the EEF, ENS, and WHG inferred ancient genetic clusters^45^ as proxies.

## Supplementary Information

Supplementary Information 1.

Supplementary Information 2.

## Data Availability

WGS for the GCAT cohort are available at EGA (https://ega-archive.org/) under extension number EGAD00001007774.

## Code availability

No software was written specifically for this project. Code use in the project can be downloaded from the following github repository: https://github.com/JorgeGarciaC/SelDem-GCAT
