# Supplementary material for: Inferring past demography and genetic adaptation in Spain using the GCAT cohort
Source: Sci Rep. 2025 Apr 24;15:14225. doi: 10.1038/s41598-025-98272-w (PMC12022144; doi:10.1038/s41598-025-98272-w)
Supplement: Supplementary file 1 — Supplementary Information 1. [file 41598_2025_98272_MOESM1_ESM.pdf]

Inferring past demography and genetic adaptation in Spain using the GCAT cohort

Jorge Garcia-Calleja[1], Simone A Biagini[1,2,3], Rafael de Cid[4,5], Francesc Calafell[1*], Elena Bosch[1*]

1. Institute of Evolutionary Biology (UPF-CSIC), Department of Medicine and Life Sciences, Universitat Pompeu Fabra, Barcelona 08003, Spain
2. Department of Archaeology and Museology, Masaryk University, Brno, Czech Republic.
3. Center of Molecular Medicine, Central European Institute of Technology, Masaryk University, Brno, Czech Republic.
4. Genomes for Life-GCAT lab, CORE Program, Germans Trias i Pujol Research Institute (IGTP), Badalona 08916, Spain.
5. Grup de REcerca en Impacte de les Malalties Cròniques i les seves Trajectòries. (GRIMTra). Germans Trias i Pujol Research Institute (IGTP), Badalona 08916, Spain

* Corresponding authors

francesc.calafell@upf.edu

elena.bosch@upf.edu

Supplementary Tables S1-S12

Table S1. Populations, groupings, and sample sizes used in ADMIXTURE, FineSTRUCTURE and GLOBETROTTER.

Table S2. Functional annotation of SNPs in the top candidate regions for selection revealed with the SDS statistic in the GCAT dataset.

Table S3. Genes in top candidate regions for selection revealed with the SDS statistic in the GCAT dataset.

Table S4. iSAFE analysis in the top candidate regions for selection revealed with the SDS statistic in the GCAT dataset.

Table S5. CLUES analysis in the top candidate SNPs for selection revealed with the SDS statistic in the GCAT dataset.

Table S6. Functional annotation of SNPs in the top candidate regions for selection revealed with the XP-EHH statistic (GCAT vs YRI) in the GCAT dataset.

Table S7. Genes in the top candidate regions for selection revealed with the XP-EHH statistic (GCAT vs YRI) in the GCAT dataset.

Table S8. iSAFE analysis in the top candidate regions for selection revealed with the XP-EHH statistic (GCAT vs YRI) in the GCAT dataset.

Table S9. CLUES analysis in the top candidate SNPs for selection revealed with the XP-EHH statistic (GCAT vs YRI) in the GCAT dataset.

Table S10. Local Ancestry Deviations (LADs) detected in the GCAT dataset when using ancient and modern DNA data from neighbouring populations.

Table S11. Geographical breakdown of the GCAT cohort.

Table S12. Samples excluded from the analyses as donors did not identify themselves (or their parents/grandparents) as white/Caucasians.

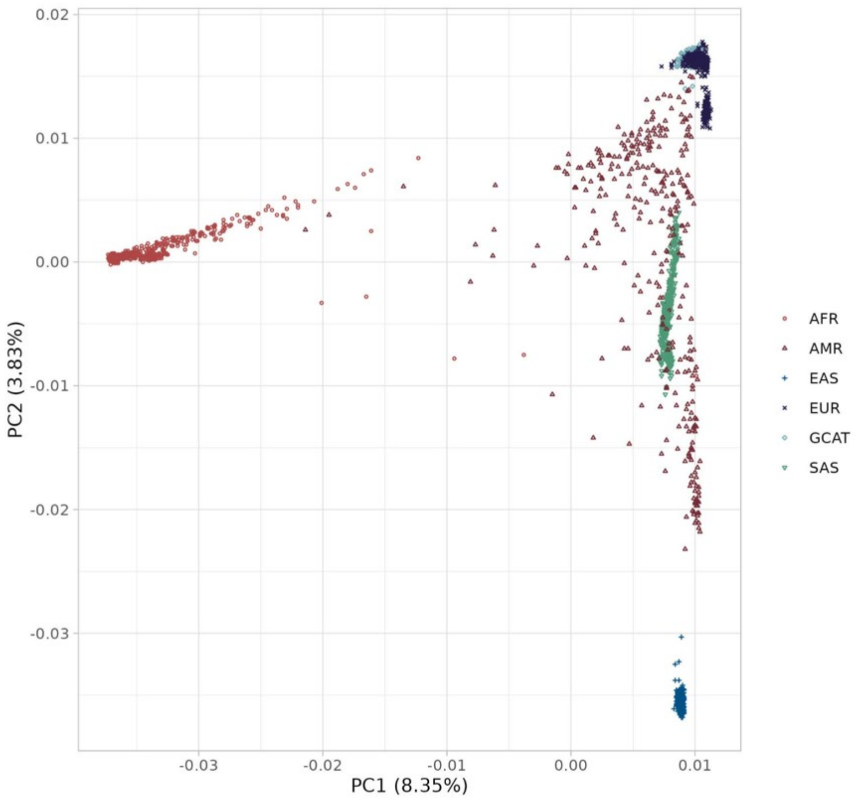

Supplementary Figure S1. **GCAT dataset within the geographical context of the 1000 GP**[1].

Principal component analysis (PCA) performed with 679,677 SNPs. Each geometric point represents an individual from a continental region. AFR, Africa; AMR, Americas; EAS, East Asia; EUR, European; SAS, South Asia.

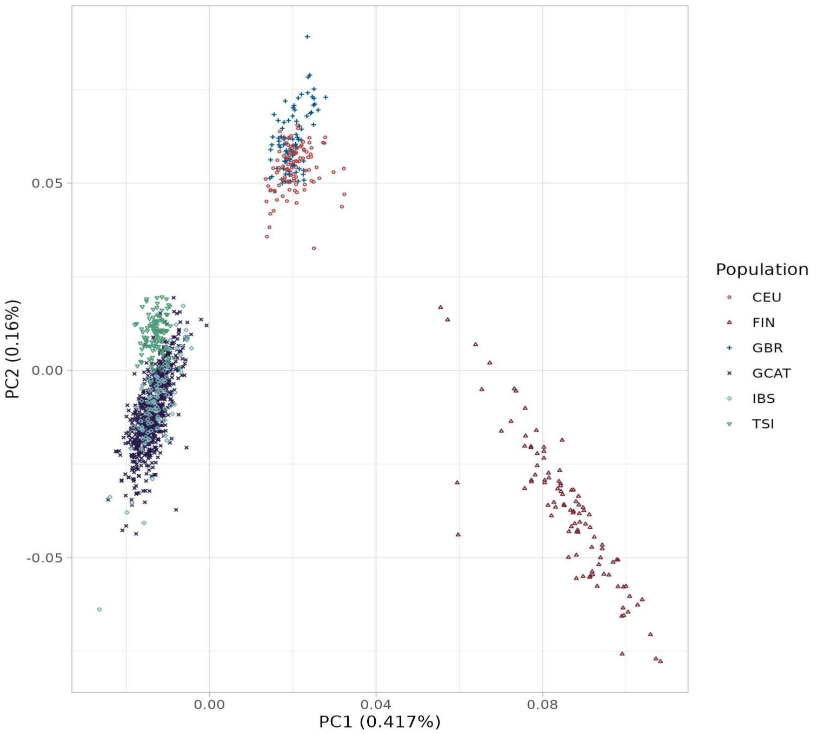

Supplementary Figure S2. GCAT dataset within the European context of the 1000 GP[1].

Principal component analysis (PCA) performed with 679,677 SNPs. Each geometric point represents an individual from a European population. CEU, Utah residents with Northern and Western European ancestry; FIN, Finnish; GBR, British; IBE, Iberian populations in Spain; TSI, Tuscans.

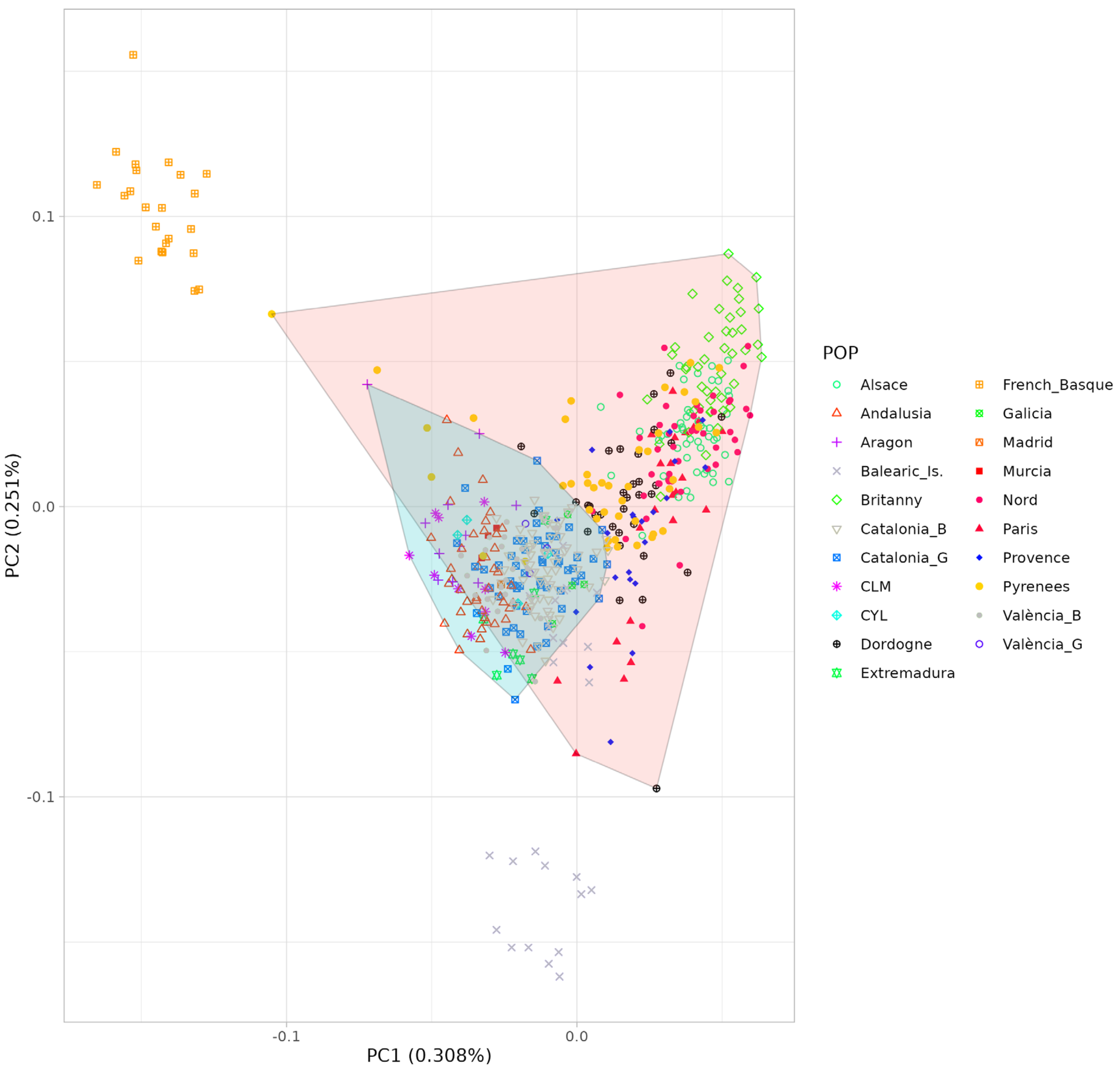

Supplementary Figure S3. GCAT dataset within the France-Spanish context. Principal component analysis (PCA) performed with 141,690 SNPs and 141 GCAT samples whose four grandparents trace their origins to the same autonomous community within Spain. The blue polygon shows the samples from the GCAT dataset[2], whereas the red polygon shows the samples from the Biagini et al. (2019) dataset[3]. See population details in Table S1.

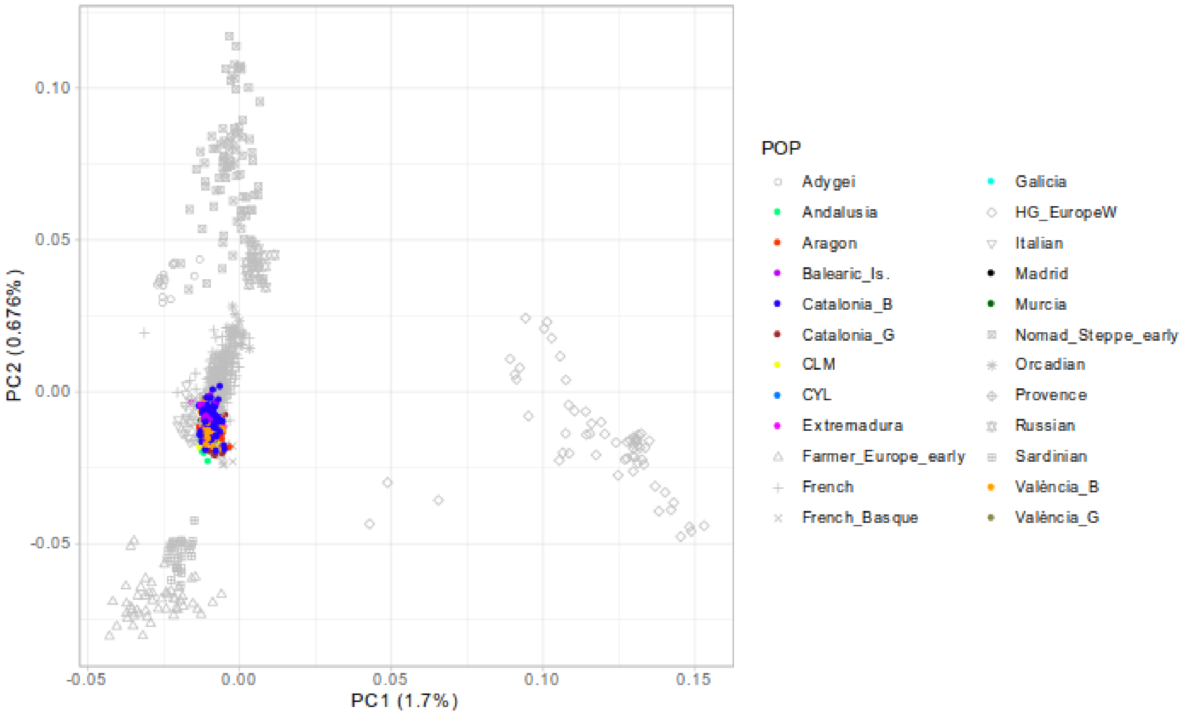

Supplementary Figure S4. GCAT dataset within the ancient European context. Principal component analysis (PCA) performed with 31,092 SNPs and 141 GCAT samples whose four grandparents trace their origins to the same autonomous community within Spain. Samples covering three ancient European components were extracted from Allentoft et al. (2024)[4]: Farmer_Europe-early, Early European Farmers (EEF); Nomad_Steppe_early, Early Nomad Steppe (ENS); HG_EuropeW, West Hunter Gatherers (WHG). See present-day population details and abbreviations in Table S1.

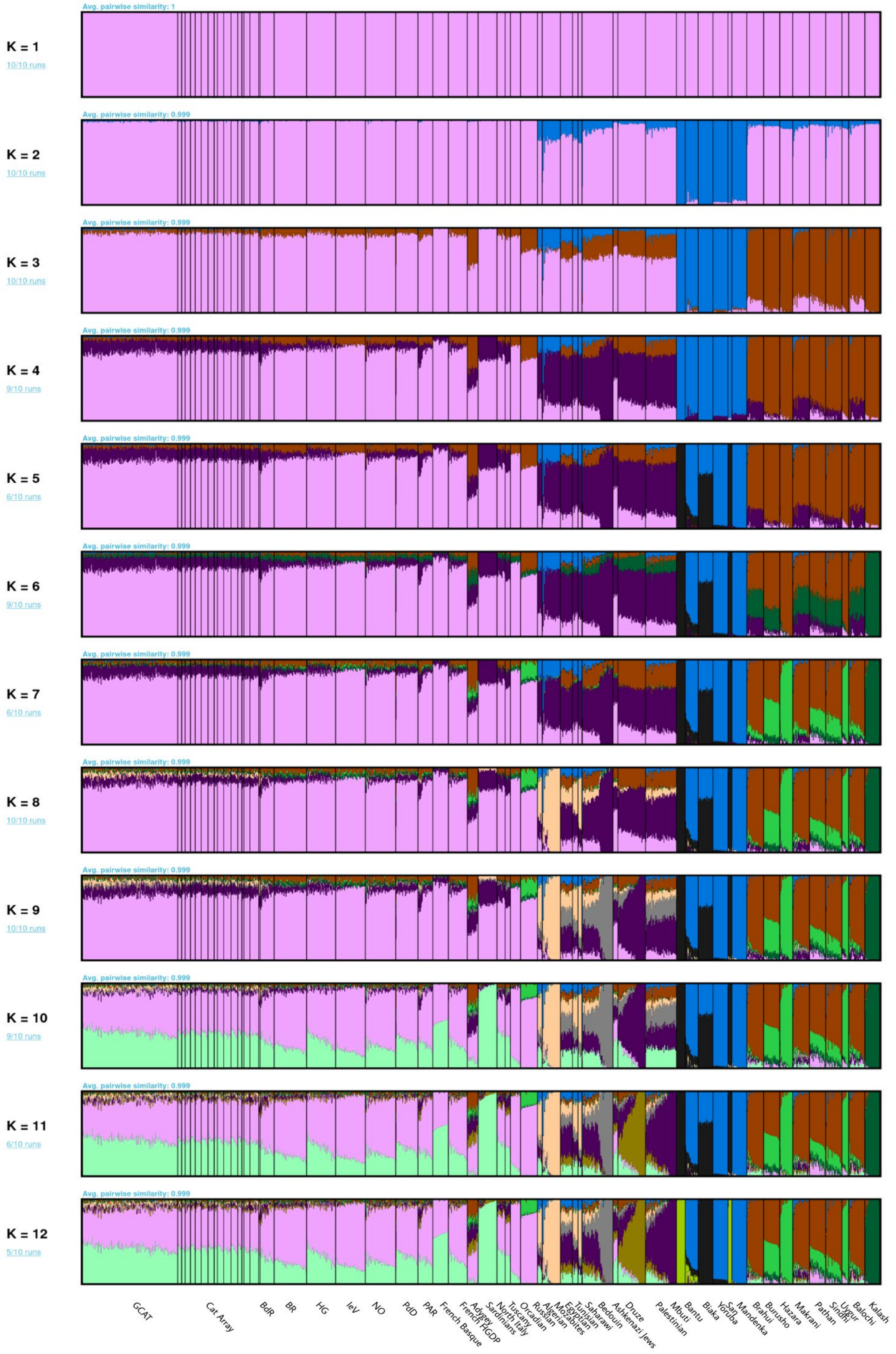

Supplementary Figure S5. GCAT dataset within the context of neighbouring populations from France and North Africa, together with African, West Asian and South Asian populations from the HGDP[5]. Lowest cross-validation errors at K=7, followed by K=8 and K=6. See references and population abbreviation details in Table S1.

A.

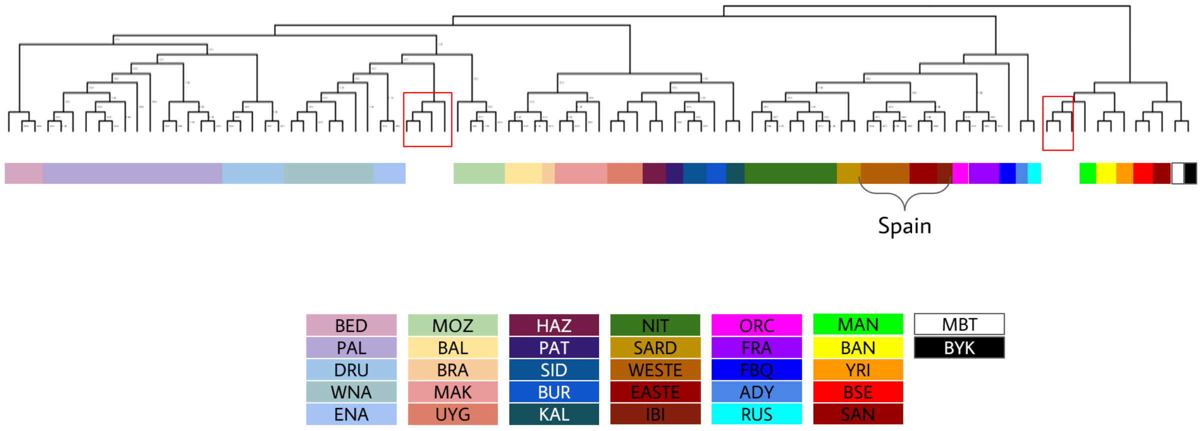

B.

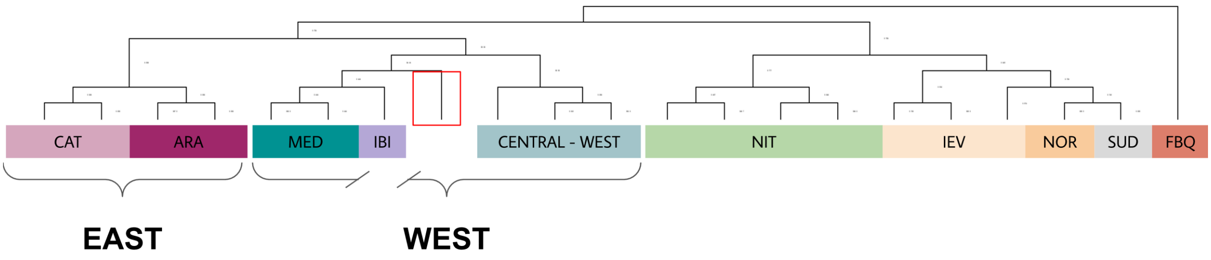

Supplementary Figure S6. FineStructure. A. Clusters detected by the fineSTRUCTURE algorithm. In most cases, clustering corresponded to geographical origin or linguistic affinity. The red boxes correspond to two clusters that are discarded due to present highly admixed individuals. In the first, beginning from the left, 10 samples from Central South Asia were discarded: Sindhi (2), Balochi (3), Brahui (2), Makrani (3). In the second, we discarded 5 samples belonging to Mozabites (2), Bantu N.E. (2) and Bedouin (1). Abbreviations: ADY, Adygei; BED, Bedouin; BAL, Balochi; BAN, Bantu; BRA, Brahui; BSE, Bantu South East Speakers; BUR, Burusho; BYK, Biaka; DRU, Druze; EASTE, East Spain; ENA, East North Africa; FBQ, French Basques; FRA, France; HAZ, Hazara; IBI, Eivissa; KAL, Kalash; MAN, Mandenka; MAK, Makrani; MOZ, Mozabites; MBT, Mbuti; NIT, North Italy; ORC, Orcadian; PAL, Palestinian; PAT, Pathan; RUS, Russian; SAN, San; SARD, Sardinian; SID, Sindhi; UYG, Uygur; WESTE, West Spain; WNA,

West North Africa; YRI, Yoruba. B. Clusters inferred from the fineSTRUCTURE output algorithm by silencing all external populations with the Force continents option. The clusters closely resemble previous results[6,7]. Abbreviations: ARA, Aragon; CAT, Catalonia; MED, Mediterranean; IBI, Eivissa; NIT, North Italy; IEV; Brittany; NOR, North France; SUD; South France; FBQ, French Basques. The North Italy Cluster (NIT) contains TSI and Northern Italy separated in two branches. The red box corresponds to a single discarded sample from Andalusia that does not cluster with other samples. For subsequent analysis, CAT and ARA clusters are merged into East Spain and MED and WEST clusters are merged into West Spain.

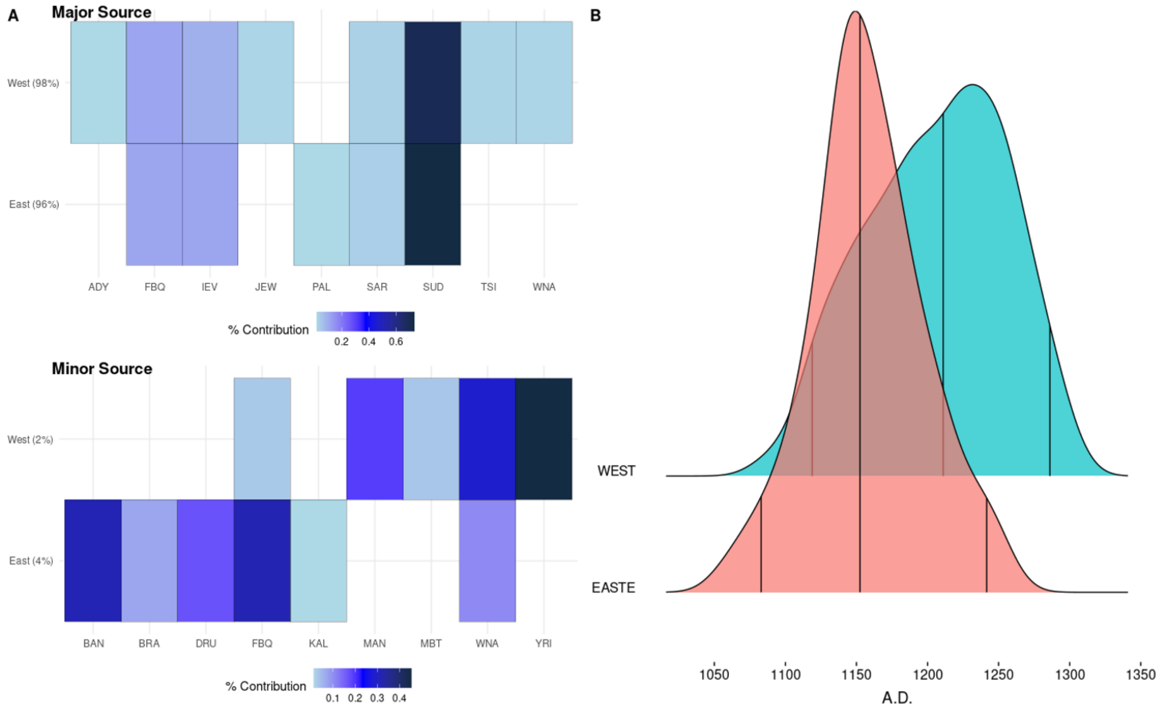

Supplementary Figure S7. Estimation of the Admixture Events. A. FastGLOBETROTTER best donor sources proportions inferred for West and East Spain clusters from low contribution to the putatively admixed population from light blue to a higher contribution for dark blue. In West Spain the mayor source accounts for a 98% of the total contribution of the admixture and the minor source 2% of the total contribution. In East Spain the mayor source accounts for a 96% of the total contribution of the admixture and the minor source 4% of the total contribution. Population abbreviations: ADY, Adygei; FBQ, French Basque; IEV, Brittany; JEW, Ashkena Jewish; PAL, Palestinian; SAR, Sardinian; SUD, South France; TSI, Tuscan; WNA, West North African; BAN, Bantu; BRA, Brahui; DRU, Druze; KAL, Kalash; MAN, Mandenka; MBT, Mbuti; YRI, Yoruba. B. Bootstrap values of the admixture dates inferred by 100 replicates with fastGLOBETROTTER for the West and East Spain clusters. Black lines indicates the mean, and the 25% and 75% quantiles.

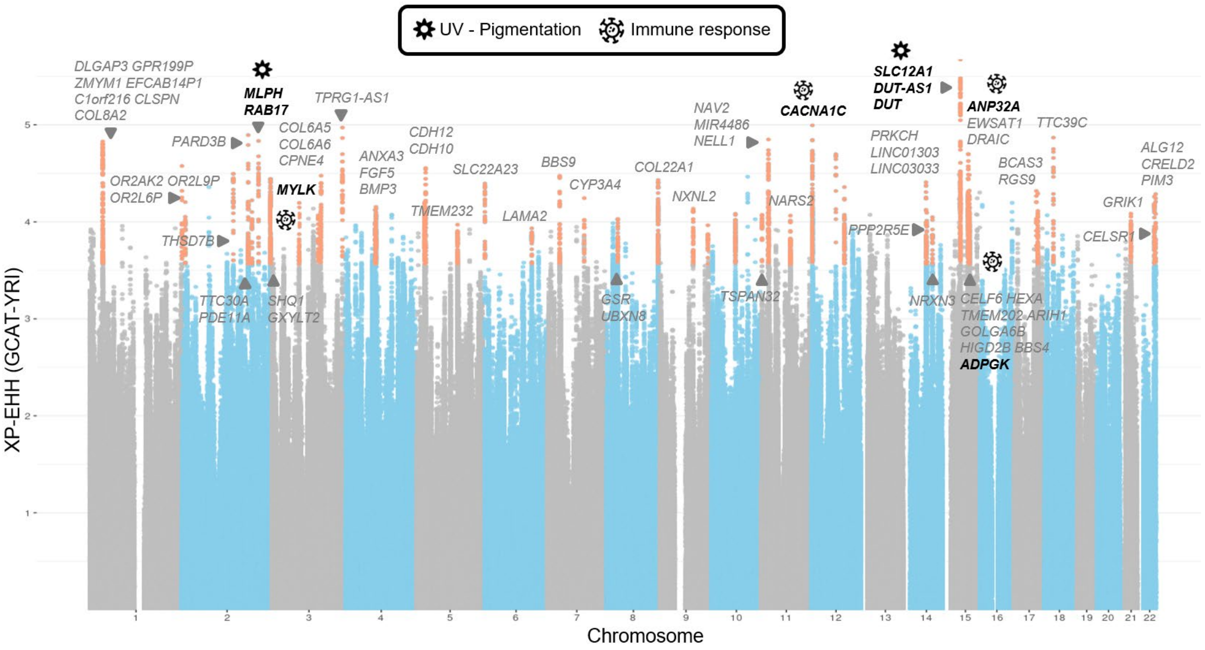

Supplementary Figure S8. Manhattan plot of signatures of positive selection in the GCAT dataset. The y-axis indicates the Cross Population Extended Haplotype Homozygosity (XP-EHH) values in the GCAT when compared versus the YRI population. Highlighted peaks indicate all the SNPs above the top 99.99% XP-EHH values that are accompanied by at least 10 SNPs above the 99.995% XP-EHH values within 1 Mb region (40 peaks in total; see details on XP-EHH values, genes, and SNP annotations in Tables S6-S9). On black bold, genes associated with plausible adaptive biological functions.

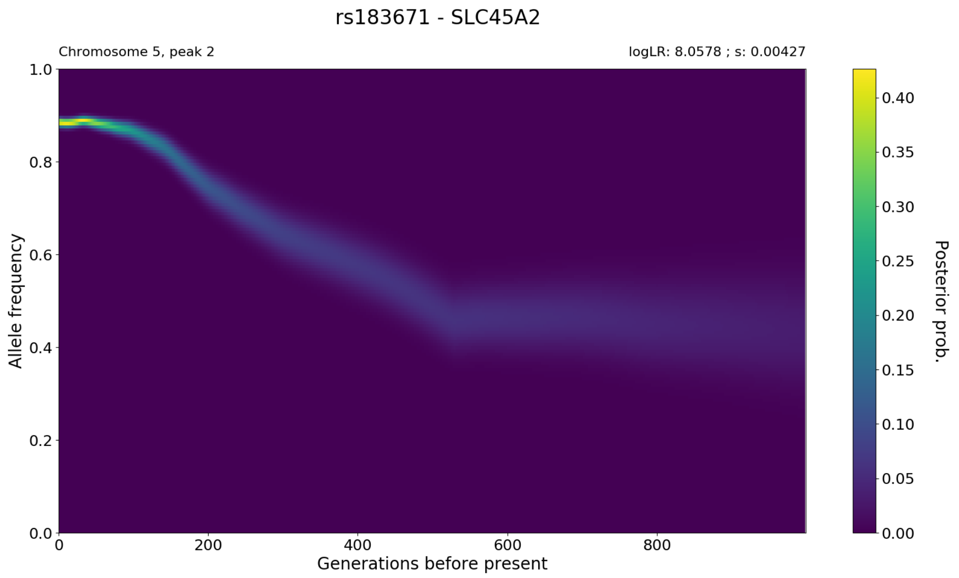

Supplementary Figure S9. Allele frequency trajectory, selection coefficient (s) and likelihood for positive selection (logLR) as obtained from CLUES for variant rs183671 within the *SLC45A2* candidate locus.

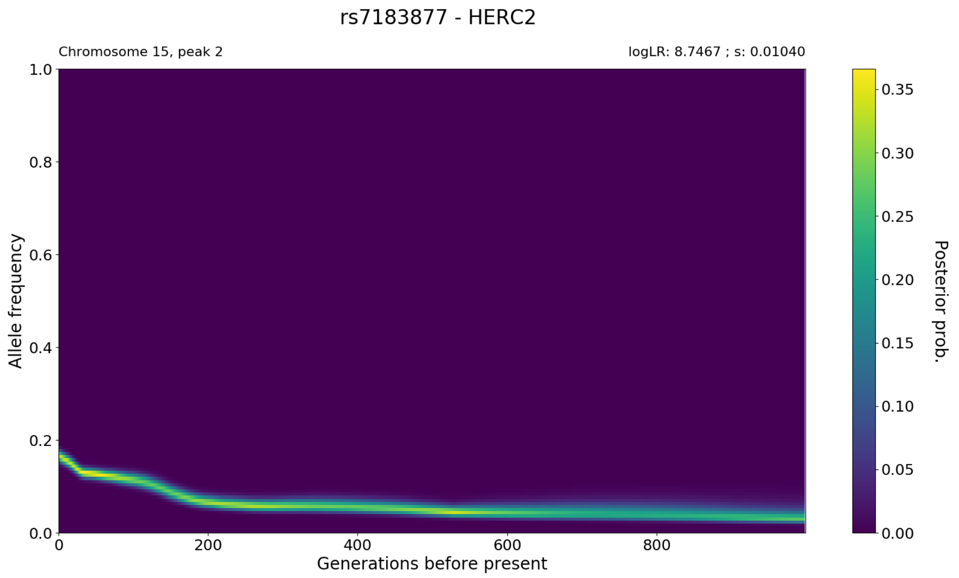

Supplementary Figure S10. Allele frequency trajectory, selection coefficient (s) and likelihood for positive selection (logLR) as obtained from CLUES for variant rs7183877 within the *OCA-HERC2* candidate locus.

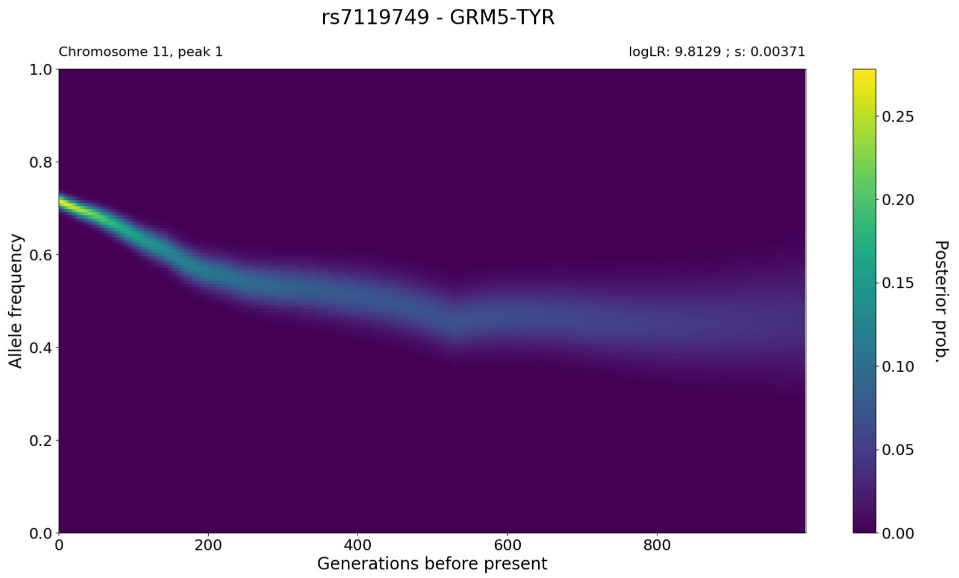

Supplementary Figure S11. Allele frequency trajectory, selection coefficient (s) and likelihood for positive selection (logLR) as obtained from CLUES for variant rs7119747 within the *GRM5-TYR* candidate region.

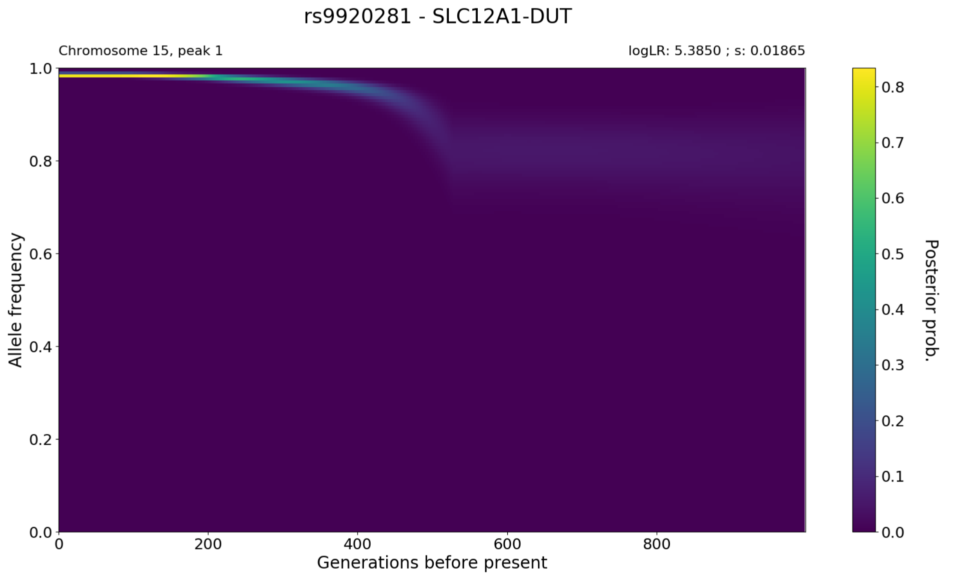

Supplementary Figure S12. Allele frequency trajectory, selection coefficient (s) and likelihood for positive selection (logLR) as obtained from CLUES for variant rs9920281 within the *SLC12A1-DUT* candidate region.

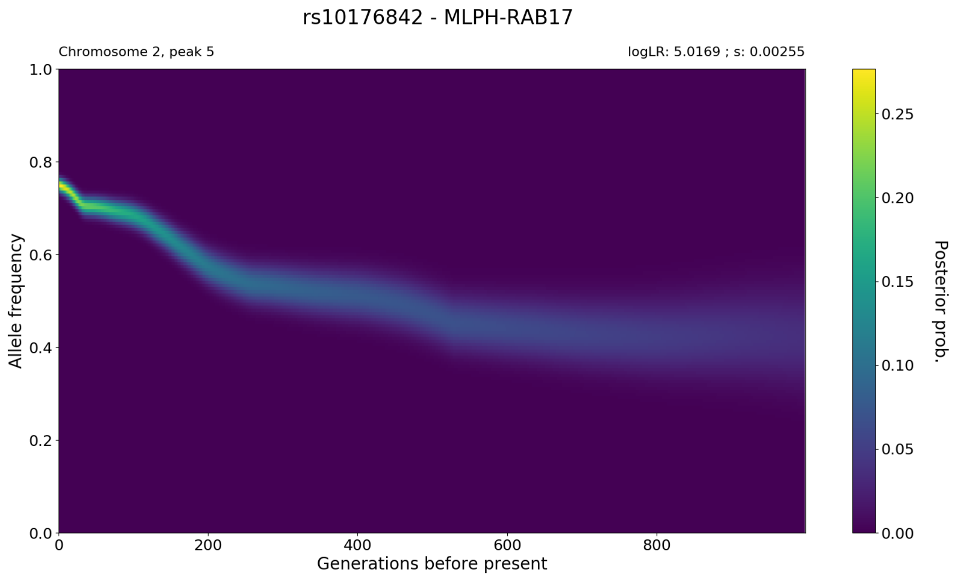

Supplementary Figure S13. Allele frequency trajectory, selection coefficient (s) and likelihood for positive selection (logLR) as obtained from CLUES for variant rs10176842 within the *MLPH-RAB17* candidate region.

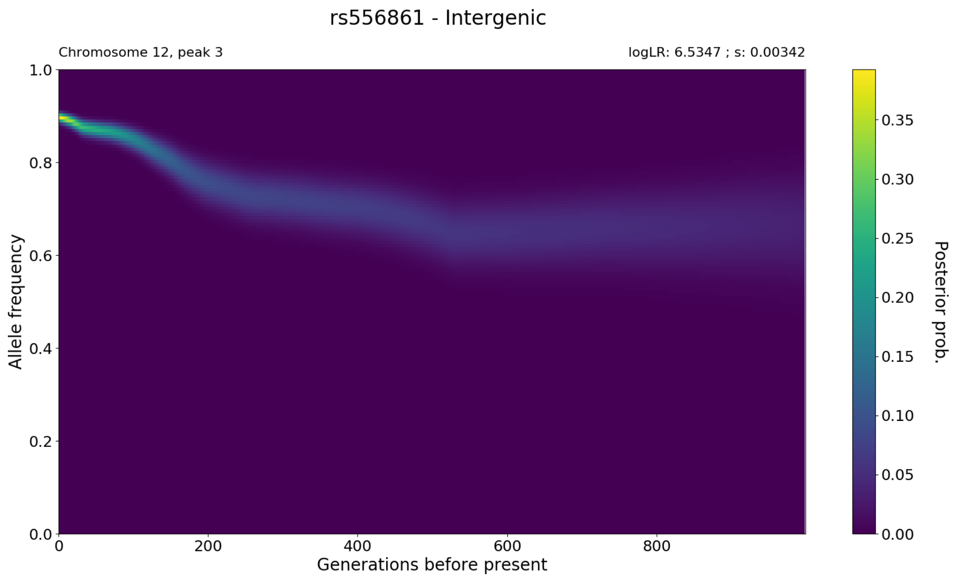

Supplementary Figure S14. Allele frequency trajectory, selection coefficient (s) and likelihood for positive selection (logLR) as obtained from CLUES for variant rs556861 within the *KITLG* candidate locus.

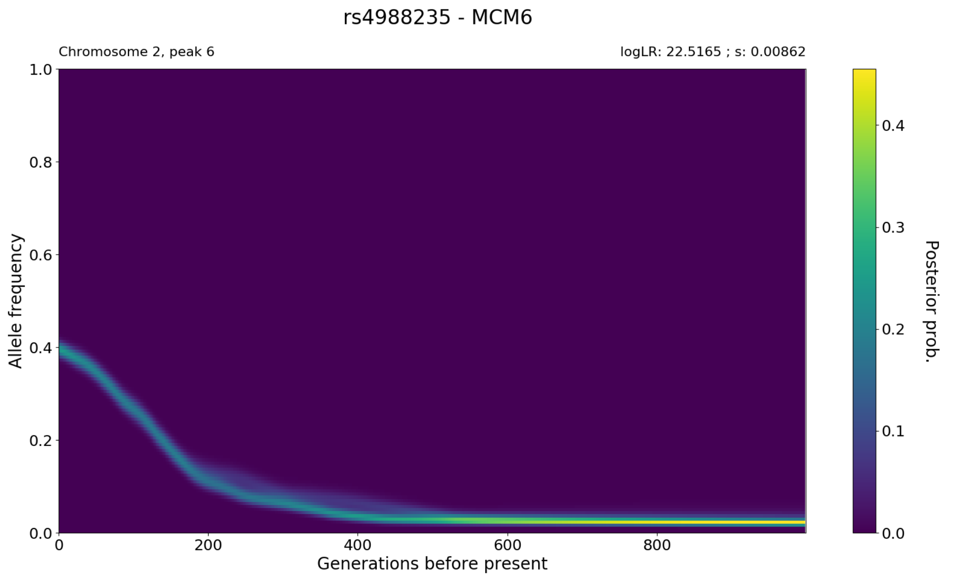

Supplementary Figure S15. Allele frequency trajectory, selection coefficient (s) and likelihood for positive selection (logLR) as obtained from CLUES for variant rs4988235 within the *MCM6 - LCT* candidate locus.

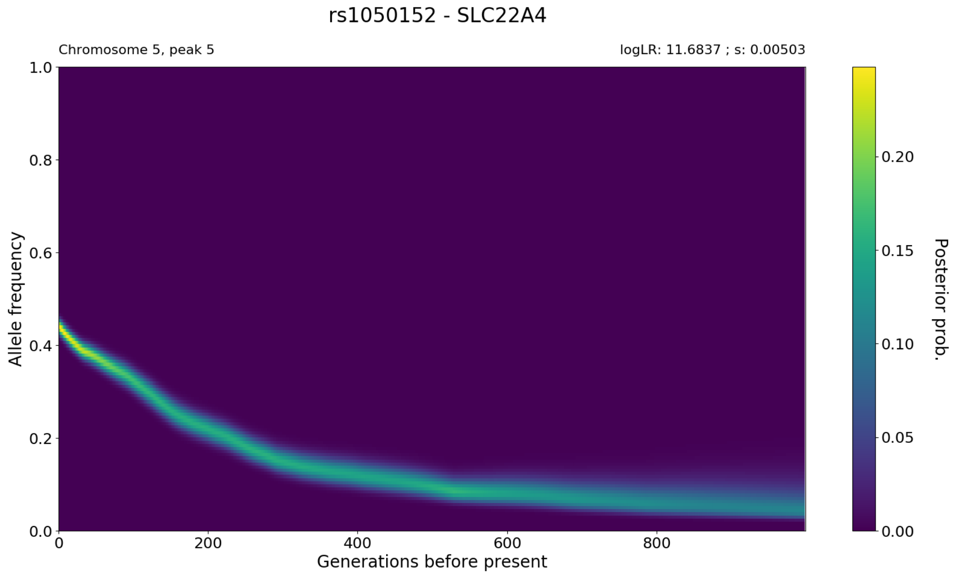

Supplementary Figure S16. Allele frequency trajectory, selection coefficient (s) and likelihood for positive selection (logLR) as obtained from CLUES for the nonsynonymous variant rs1050152 within the *SLC22A4* candidate locus.

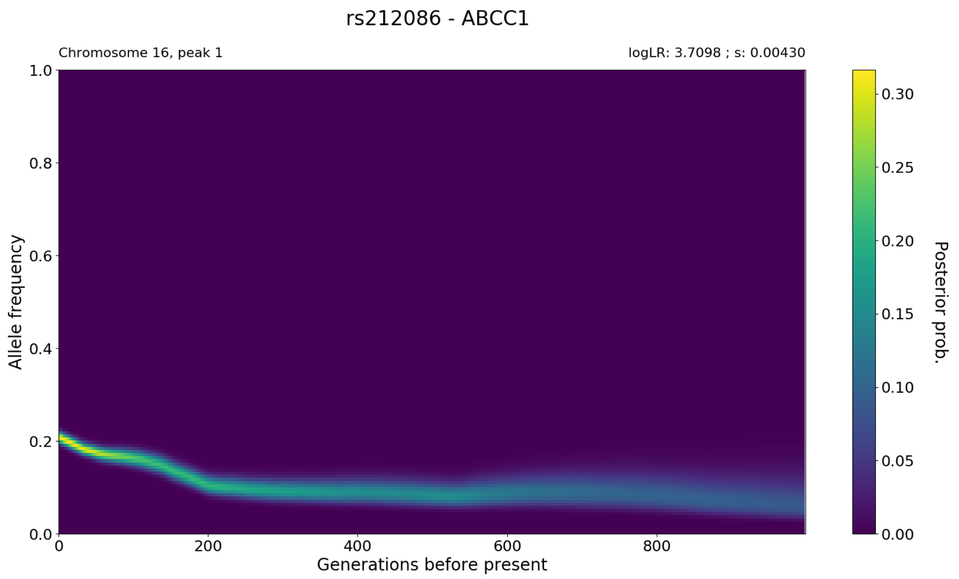

Supplementary Figure S17. Allele frequency trajectory, selection coefficient (s) and likelihood for positive selection (logLR) as obtained from CLUES for variant rs212086 within the *ABCC1* candidate locus.

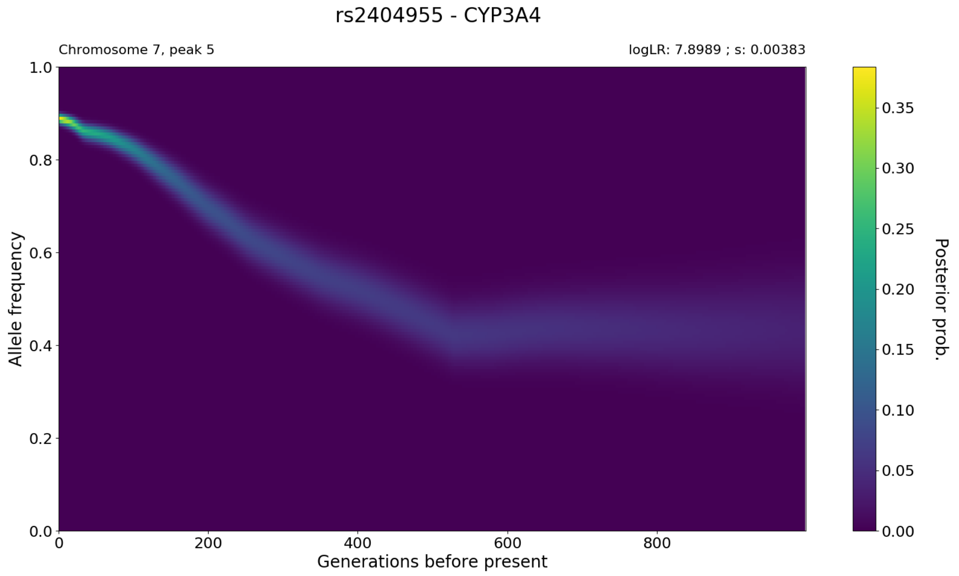

Supplementary Figure S18. Allele frequency trajectory, selection coefficient (s) and likelihood for positive selection (logLR) as obtained from CLUES for variant rs2404955 within the *CYP3A4* candidate locus.

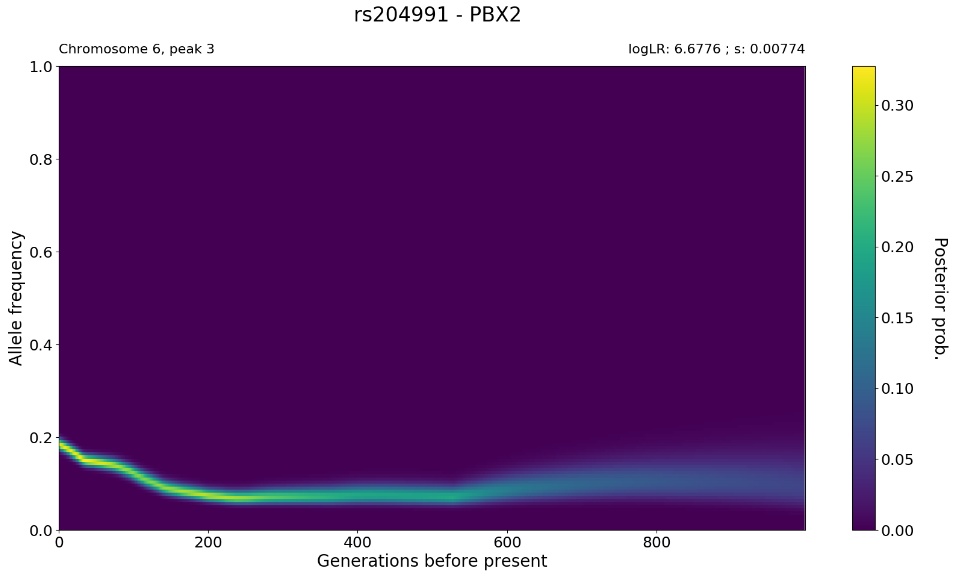

Supplementary Figure S19. Allele frequency trajectory, selection coefficient (s) and likelihood for positive selection (logLR) as obtained from CLUES for variant rs204991 within the *PBX2* candidate locus.

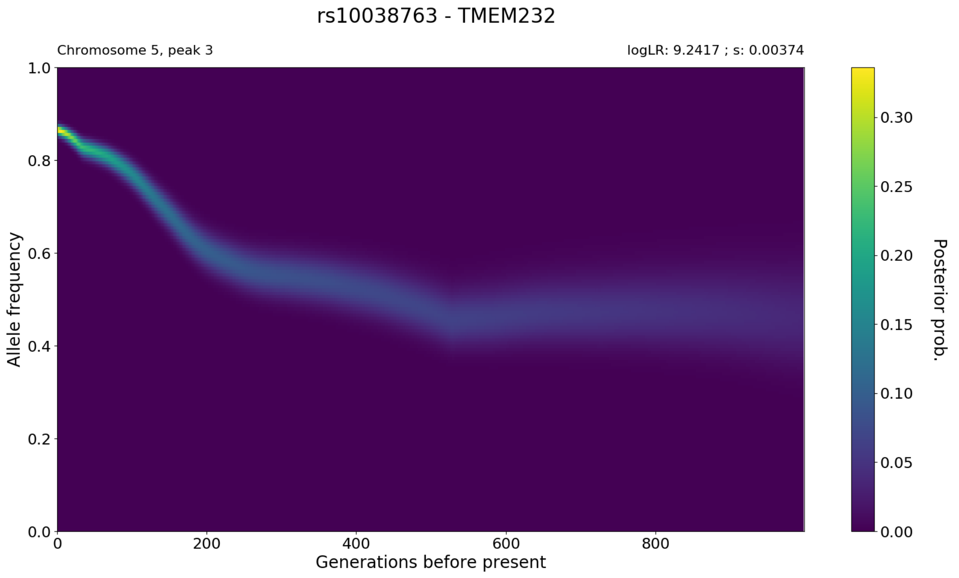

Supplementary Figure S20. Allele frequency trajectory, selection coefficient (s) and likelihood for positive selection (logLR) as obtained from CLUES for variant rs10038763 within the *TMEM232* candidate locus.

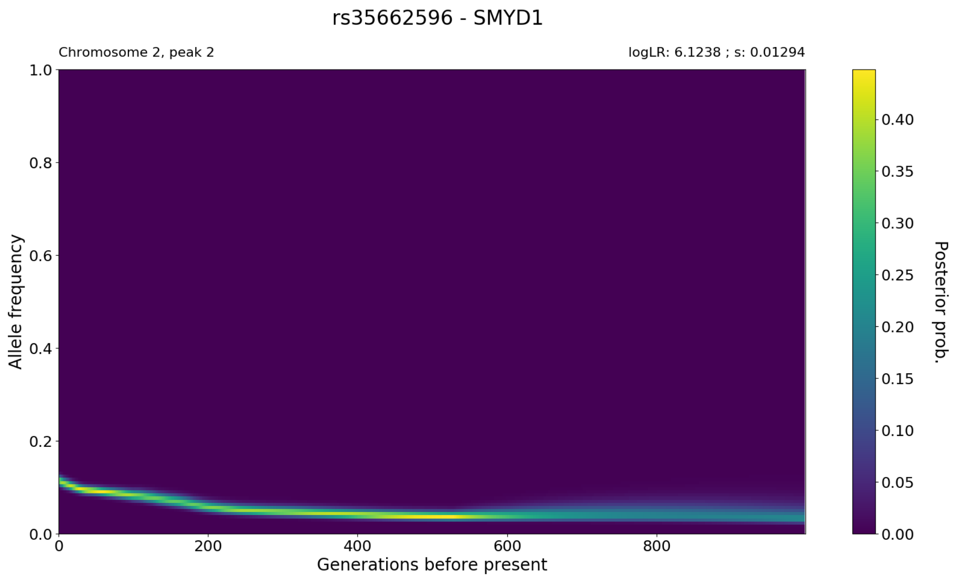

Supplementary Figure S21. Allele frequency trajectory, selection coefficient (s) and likelihood for positive selection (logLR) as obtained from CLUES for variant rs35662596 within the candidate *SMYD1* locus.

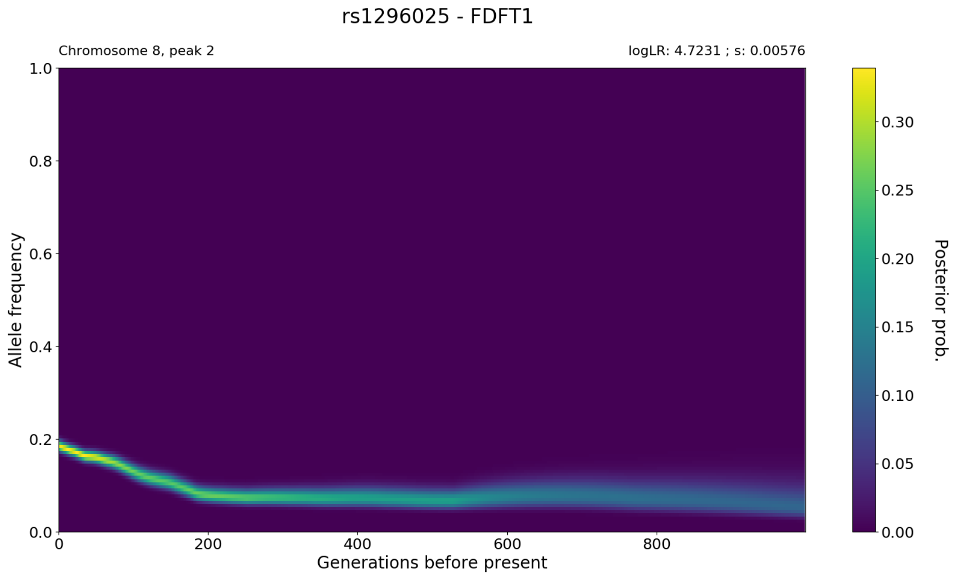

Supplementary Figure S22. Allele frequency trajectory, selection coefficient (s) and likelihood for positive selection (logLR) as obtained from CLUES for variant rs1296025 within the *FDFT1* candidate locus.

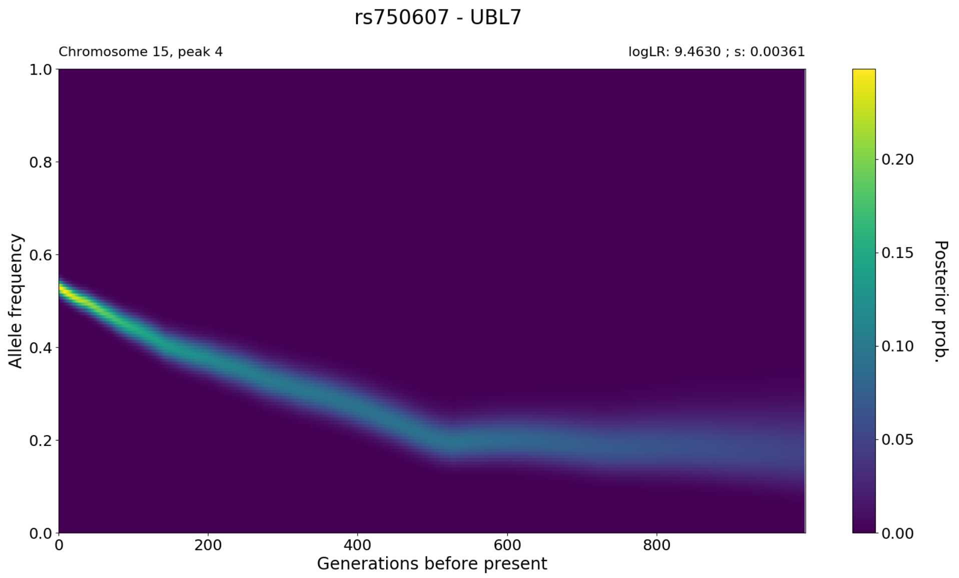

Supplementary Figure S23. Allele frequency trajectory, selection coefficient (s) and likelihood for positive selection (logLR) as obtained from CLUES for variant rs750607 within the *UBL7* candidate locus.

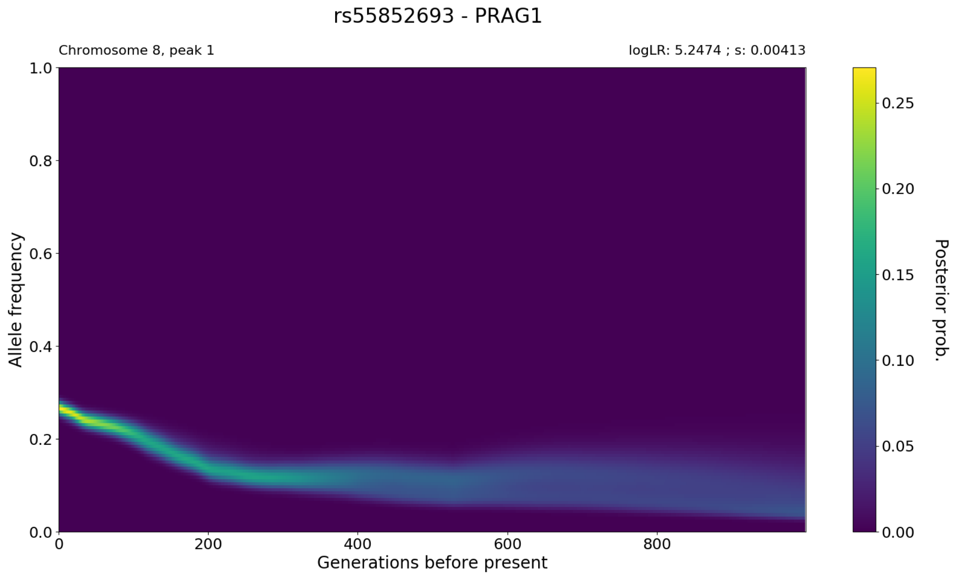

Supplementary Figure S24. Allele frequency trajectory, selection coefficient (s) and likelihood for positive selection (logLR) as obtained from CLUES for variant rs55852693 within the *PRAG1* candidate locus.

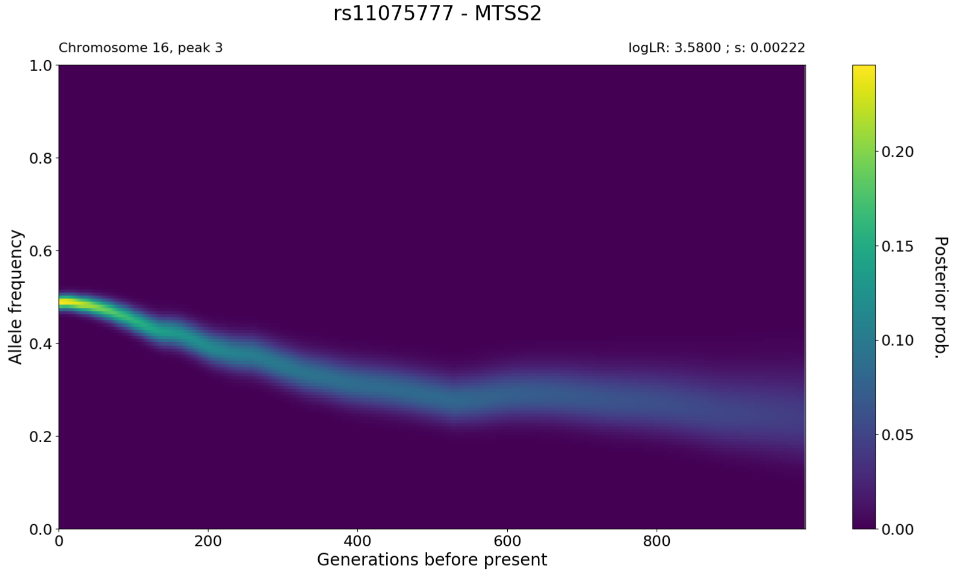

Supplementary Figure S25. Allele frequency trajectory, selection coefficient (s) and likelihood for positive selection (logLR) as obtained from CLUES for variant rs11075777 within the *MTSS2* candidate locus.

A.

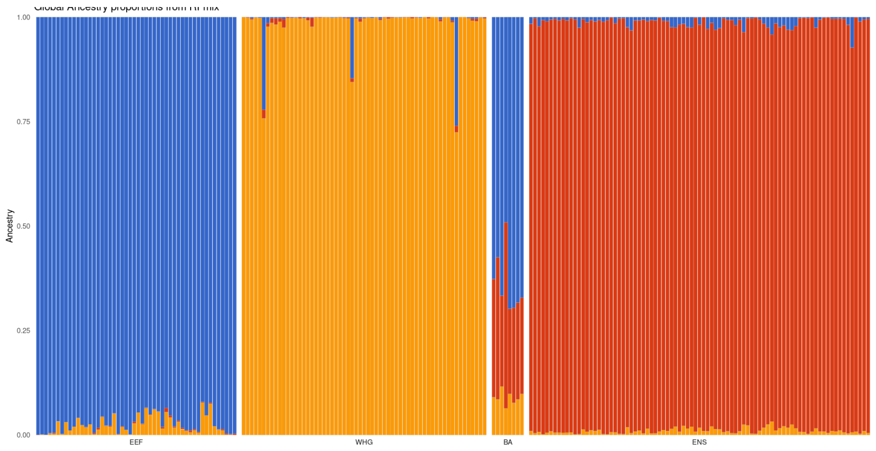

B.

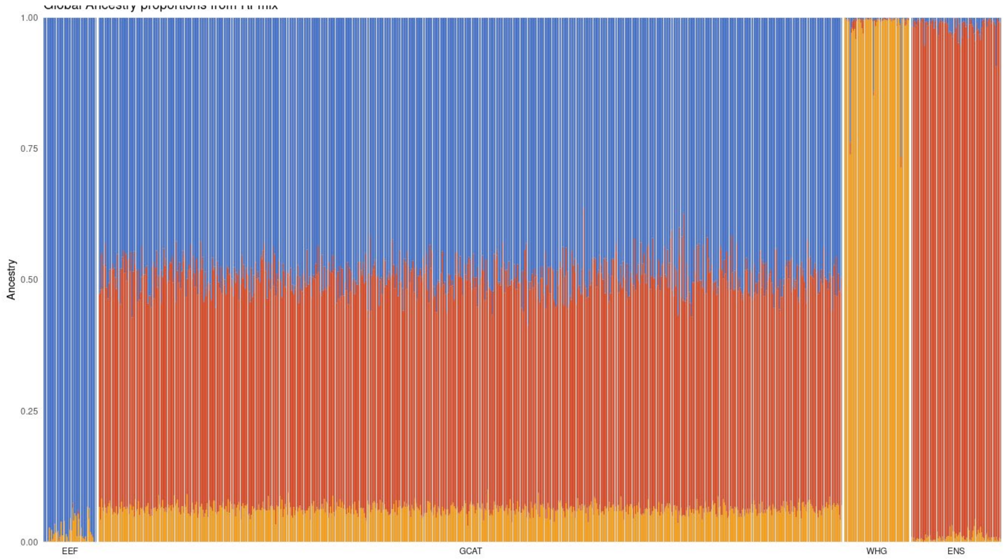

Supplementary Figure S26. GCAT data in the context of ancient data. A. Global ancient ancestry proportions as obtained from RFmix using phased imputed samples from population genomics data of post-glacial western Eurasia (EEF, Early European Farmers; WHG, West Hunter Gatherers; BA, Bronze Age; and ENS, Early Nomad Steppes) publicly available[4]. **B.** Global ancient ancestry proportions obtained from RFmix in the GCAT dataset using the three main ancient components; BA were not included as a reference since, as seen in panel A, they are themselves an admixed population

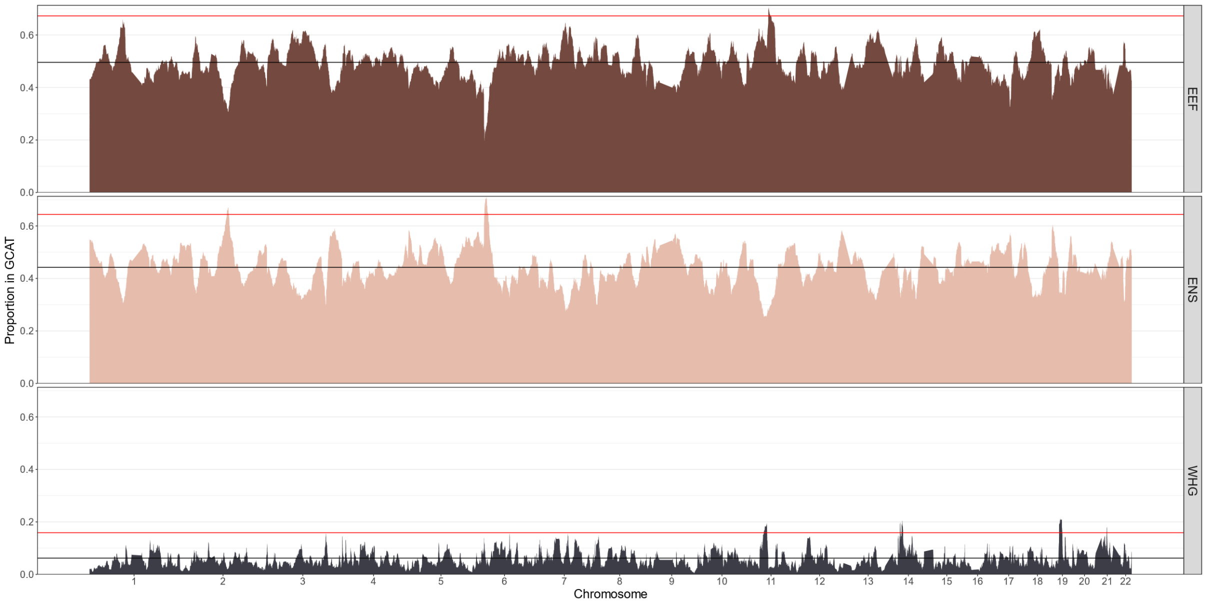

Supplementary Figure S27. Inference of external ancestry components and search for Local Ancestry Deviations (LAD). Ancient ancestry proportions across chromosomes as obtained from RFmix using phased imputed samples from population genomics data of post-glacial western Eurasia (EEF, Early European Farmers; WHG, West Hunter Gatherers; BA, Bronze Age; and ENS, Early Nomad Steppes) publicly available[4]. The black lines show the average proportion for each ancestry. The red lines indicate 3 standard deviations above the genomic mean. Regions above the line were considered locally deviated. See details in Table S10.

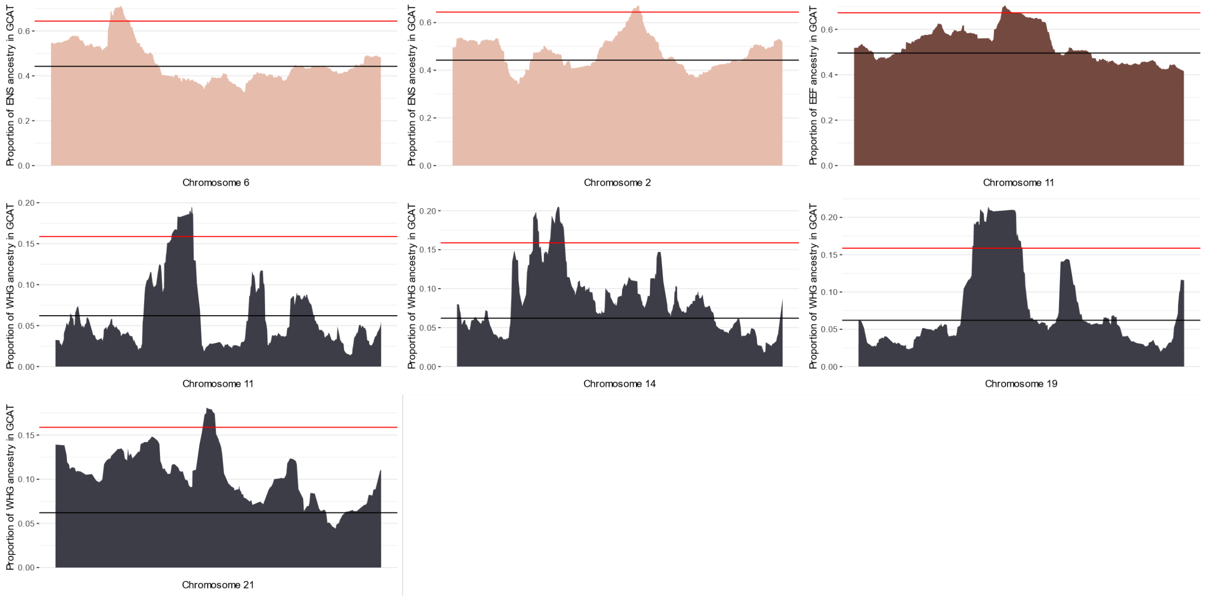

Supplementary Figure S28. Regions enriched for specific ancestry components: Details of all the deviated regions obtained from RFmix using phased imputed samples from population genomics data of post-glacial western Eurasia (EEF, Early European Farmers; WHG, West Hunter Gatherers; BA, Bronze Age; and ENS, Early Nomad Steppes) publicly available[4]. Black line shows the average proportion for each ancestry. The red line shows 3 standard deviations above the ancestry genomic mean, which is the threshold for local ancestry deviation.

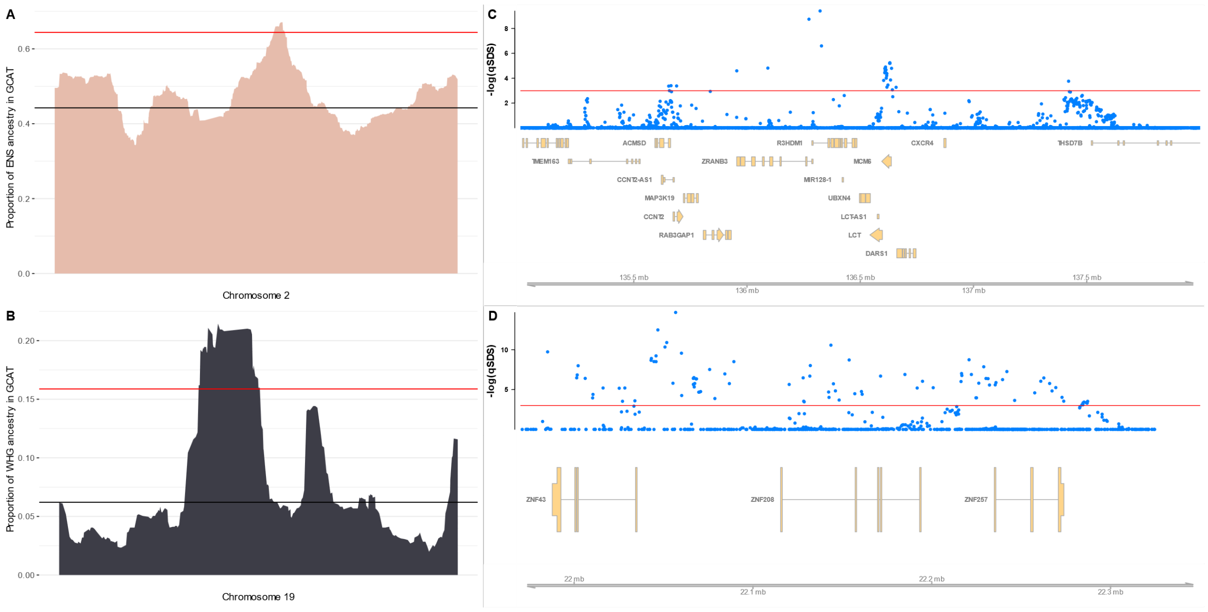

Supplementary Figure S29. Post-admixture selection in LCT and zinc finger genes.

Ancestral proportions in the GCAT dataset for **A.** the ENS ancestry in the LAD region overlapping the *LCT-MCM6* region in chromosome 2 and **B.** the WHG ancestry in the LAD region overlapping a cluster of zinc finger protein genes in chromosome 19. The black line shows the mean proportion for each ancestry. The red line indicates three standard deviations above the genomic mean. **C.** Plot showing the transformed SDS scores after FDR correction and the corresponding genes underlying the LAD region show in A. **D.** Plot showing the transformed SDS scores after FDR correction and the candidate genes underlying the LAD region show in B. The red line shows the 0.05 statistical significance threshold. See details in Table S10.

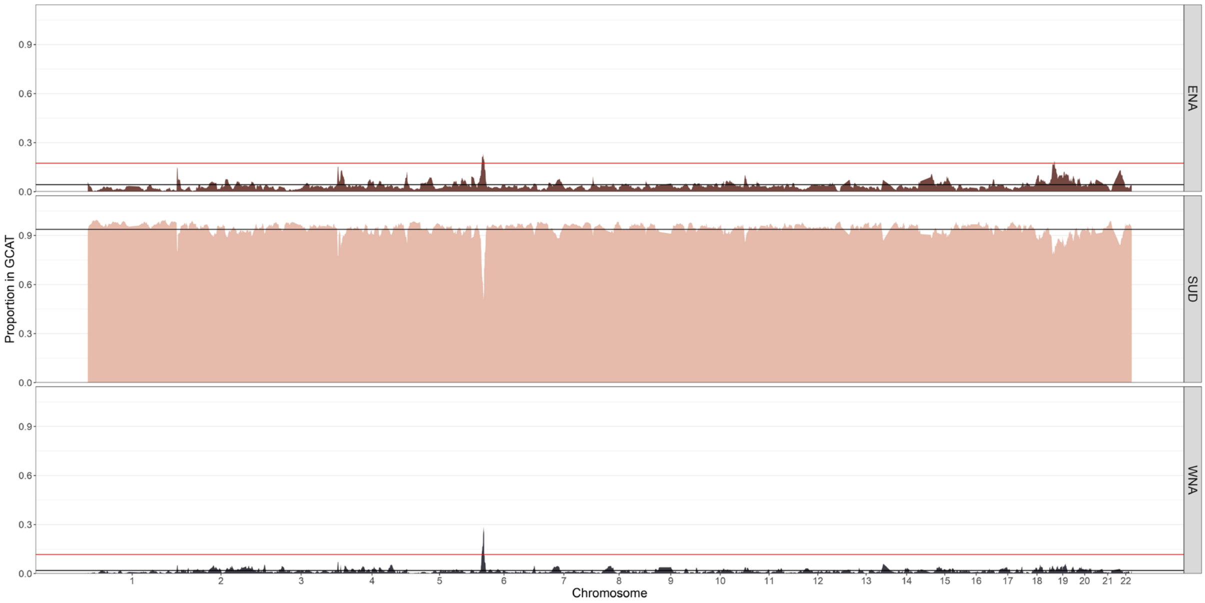

Supplementary Figure S30. Inference of external modern ancestry components and search for Local Ancestry Deviations (LAD). Modern ancestry proportions across chromosomes as obtained from RFmix using phased samples from neighboring populations[8,9] (See details in Table S1). The black lines show the average proportion for each ancestry. The red lines indicate 4.42 standard deviations above the genomic mean. Regions above the line we considered locally deviated.

References

1. Byrska-Bishop, M. *et al.* High-coverage whole-genome sequencing of the expanded 1000 Genomes Project cohort including 602 trios. *Cell* **185**, 3426-3440.e19 (2022).
2. Valls-Margarit, J. *et al.* GCAT|Panel, a comprehensive structural variant haplotype map of the Iberian population from high-coverage whole-genome sequencing. *Nucleic Acids Res* **50**, 2464–2479 (2022).
3. Biagini, S. A. *et al.* People from Ibiza: an unexpected isolate in the Western Mediterranean. *Eur J Hum Genet* **27**, 941–951 (2019).
4. Allentoft, M. E. *et al.* Population genomics of post-glacial western Eurasia. *Nature* **625**, 301–311 (2024).
5. Bergström, A. *et al.* Insights into human genetic variation and population history from 929 diverse genomes. *Science* **367**, eaay5012 (2020).
6. Bycroft, C. *et al.* Patterns of genetic differentiation and the footprints of historical migrations in the Iberian Peninsula. *Nat Commun* **10**, 551 (2019).
7. Hernández, C. L. *et al.* Human Genomic Diversity Where the Mediterranean Joins the Atlantic. *Mol Biol Evol* **37**, 1041–1055 (2020).
8. Biagini, S. A., Ramos-Luis, E., Comas, D. & Calafell, F. The place of metropolitan France in the European genomic landscape. *Hum Genet* **139**, 1091–1105 (2020).
9. Lazaridis, I. *et al.* Genomic insights into the origin of farming in the ancient Near East. *Nature* **536**, 419–424 (2016).
